# *SiSTL1*, encoding a large subunit of ribonucleotide reductase, is crucial for plant growth, chloroplast biogenesis, and cell cycle progression in *Setaria italica*

**DOI:** 10.1093/jxb/ery429

**Published:** 2018-12-07

**Authors:** Chanjuan Tang, Sha Tang, Shuo Zhang, Mingzhao Luo, Guanqing Jia, Hui Zhi, Xianmin Diao

**Affiliations:** Institute of Crop Sciences, Chinese Academy of Agricultural Sciences, Beijing, China

**Keywords:** Cell cycle progression, chloroplast biogenesis, DNA replication, growth retardation, ribonucleotide reductase, *SiSTL1*, striped leaf

## Abstract

The activity of ribonucleotide reductase (RNR), which catalyses the transformation of four ribonucleoside diphosphates (NDPs) to their corresponding deoxyribonucleoside diphosphates (dNDPs), is the main determiner of the cellular concentration of dNTP pools and should be tightly coordinated with DNA synthesis and cell-cycle progression. Constitutively increased or decreased RNR activity interferes with DNA replication and leads to arrested cell cycle progression; however, the mechanisms underlying these disruptive effects in higher plants remain to be uncovered. In this study, we identified a RNR large subunit mutant, *sistl1*, in *Setaria italica* (foxtail millet), which exhibited growth retardation as well as striped leaf phenotype, i.e. irregularly reduced leaf vein distances and decreased chloroplast biogenesis. We determined that a Gly_737_ to Glu substitution occurring in the C-terminus of the SiSTL1 protein slightly affected its optimal function, leading in turn to the reduced expression of genes variously involved in the assembly and activation of the DNA pre-replicative complex, elongation of replication forks and S phase entry. Our study provides new insights into how *SiSTL1* regulates plant growth, chloroplast biogenesis, and cell cycle progression in Poaceae crops.

## Teaser

A ribonucleotide reductase large subunit mutant isolated from *Setaria italica* had a Gly_737_ to Glu substitution in the C-terminus of the protein and exhibited growth retardation and striped leaves.

## Introduction

The cellular concentration of deoxyribonucleoside triphosphate (dNTP) pools fluctuates with cell cycle progression (Chabes and Stillman, 2007). Correct levels of dNTP pools are critical for the accomplishment and high fidelity of DNA replication (Poli *et al.*, 2012). The level of dNTP pools is generally restricted during the G_1_ phase. Upon entry into the S phase, the concentration increases sharply—by approximately 3-fold in *Saccharomyces cerevisiae*—and then drops to the same levels as in G1 phase during the G_2_ and M phases (Chabes *et al.*, 2003; Poli *et al.*, 2012). dNTPs are generated by two pathways. In the *de novo* synthesis pathway, presumed to be the main biosynthetic pathway, dNTPs are synthesized from simple substances such as ribose phosphate, amino acids, and CO. In the second pathway, termed the salvage pathway, dNTPs are generated by simple transfer reactions involving deoxyribose phosphates derived from the *de novo* synthesis pathway. The activity of ribonucleotide reductase (RNR), which catalyses the transformation of NDPs to their corresponding dNDPs in the *de novo* synthesis pathway, is the main determiner of cellular dNTP-pool concentrations and is tightly coordinated with DNA replication, cell cycle progression, and DNA repair (Chabes *et al.*, 2003; Poli *et al.*, 2012).

Most eukaryotic RNRs are α_2_β_2_ heterotetramers comprising two large subunits (R1/RNRL) and two small subunits (R2/RNRS) (Nordlund and Reichard, 2006; Reichard, 2010; Sanvisens *et al.*, 2013). The R1 subunit contains both catalytic and allosteric regulation domains, while R2 contains a non-heme dinuclear iron center. During each reduction reaction, a stable tyrosyl radical is created and transferred to the catalytic cysteine pair of R1 (Cys_218_ and Cys_443_ in *S. cerevisiae*) (Kolberg *et al.*, 2004). The catalytic cysteine pair is then converted from the reduced form to the oxidized (disulfide-bonded) form. The disulfide-bond is subsequently reduced by thioredoxin and glutaredoxin to regenerate active R1 (reduced state) for the next catalytic cycle (Kolberg *et al.*, 2004). A conserved cysteine pair at the R1 C-terminal end (designated the CX_2_C motif in eukaryotic R1s) is indispensable for the regeneration of the R1 catalytic cysteine pair, as it mediates the interaction of this cysteine pair and thioredoxin/glutaredoxin. In addition to the CX_2_C motif, the last ~100 amino acids located before the CX_2_C motif at the R1 C-terminus, designated the C-terminal insertion (CI) region, is also important for optimal R1 activity (Zhang *et al.*, 2007).

Because the cellular dNTP pool is sufficient for only a small fraction of DNA replication, up-regulation of RNR activity is necessary when cells enter the S phase or experience DNA damage (Poli *et al.*, 2012). Strategies used by cells for RNR up-regulation include the transcriptional induction of RNR genes, degradation of RNR inhibitors, and subcellular redistribution of RNR subunits (Nordlund and Reichard, 2006; Sanvisens *et al.*, 2013). The E2F family of transcription factors, which is regulated in a cell cycle-dependent manner, plays a central role in controlling the expression of genes required for cell cycle progression, particularly DNA synthesis (Stevens and La Thangue, 2003). Up-regulation of both RNRL and RNRS during the S phase is mediated by E2F transcription factors. In human and mouse cells, R1 levels are almost constant and are present in excess during the cell cycle (Chabes *et al.*, 2004; Shao *et al.*, 2006). S-phase-specific RNR activity is determined by the E2F-dependent cell cycle regulation of R2 genes (Chabes *et al.*, 2004). Other studies, however, have shown that R1 genes also exhibit S-phase-specific expression mode and are regulated by MBF/E2F transcription factors in *S. cerevisiae* and tobacco (Chabouté *et al.*, 2002; Chabes *et al.*, 2004; Lincker *et al.*, 2004; Sanvisens *et al.*, 2013). In addition to this transcriptional regulation, RNR activity is controlled by the Mec1/Rad53 protein kinase-dependent proteolysis of Sml1 (Zhao *et al.*, 2000, 2001). The concentration of the Sml1 protein, a RNR large subunit inhibitor, also fluctuates during the cell cycle and is lowest during the S phase (Zhao *et al.*, 2001). During G_0_ and G_1_ phases, Sml1 competitively combines with the catalytic site of the R1 subunit and thus blocks the reduction activity of R1 (Zhang *et al.*, 2007). When cells enter the S phase or encounter DNA replication stress, Sml1 is phosphorylated and degraded in a Mec1/Rad53-dependent manner, thereby relieving RNR inhibition. To summarize, one conserved theme of RNR activity, albeit controlled by different mechanisms, is that it is cell cycle regulated, restricted during G_0_ and G_1_ phases and peaking at the S phase (Nordlund and Reichard, 2006; Guarino *et al.*, 2014).

DNA synthesis begins with the assembly and activation of replication origins (Sheu *et al.*, 2016). During this process, a double hexameric minichromosome maintenance (MCM) complex, composed of two Mcm2–Mcm7 hexamers, is loaded onto the replication origin to form a pre-replicative complex (pre-RC) with the help of an origin recognition complex (ORC) and the licensing factors Cdc6 and Cdt1 (Das *et al.*, 2015; Sheu *et al.*, 2016). Cdc45 is then recruited to activate the MCM complex with the assistance of S-phase cyclin-dependent kinases (CDKs) and Dbf4-dependent Cdc7 kinase (Rossbach *et al.*, 2017). After these two steps, the replication origins are fully activated, which enables the recruitment of DNA polymerase and other replisome components to form the replication forks needed to start DNA elongation (Sheu *et al.*, 2016).

Because RNR activity and DNA replication are interconnected, much research has been performed to explain how disruption of RNR activity impedes DNA replication and cell cycle progression (Chabes and Stillman, 2007; Poli *et al.*, 2012; Giannattasio and Branzei, 2017). One proposed mechanism, conserved among budding yeast, fission yeast and human cells, is that disruption of RNR activity activates the S-phase checkpoint, which subsequently delays S-phase entry, increases dNTP synthesis and prevents late replication-origin firing (Giannattasio and Branzei, 2017). The S-phase checkpoint, known as the Mec1/Rad53 pathway in budding yeast and the ATR–CHK1 pathway in human cells, is composed of multiple serine/threonine kinases (Sun *et al.*, 1995; Guo *et al.*, 2000). In human cells, ATR–CHK1-mediated phosphorylation events inhibit the CDK activators Cdc25A, Cdc25B, and Cdc25C, thereby inhibiting the activities of CDK2–cyclin A/E and CDK1–cyclin B to delay S-phase entry (Krek *et al.*, 1995; Giannattasio and Branzei, 2017). In addition, ATR can induce dNTP production by up-regulating E2F1. Late replication-origin firing is prevented by the phosphorylation events of ATR–CHK1 on proteins required for replication fork formation, such as MCM2, RPA2, ExoI, and BLM (Giannattasio and Branzei, 2017). Furthermore, disturbed RNR activity can impede DNA replication and cell cycle progression by mechanisms independent of the Mec1/Rad53 pathway. In *S. cerevisiae*, continuous induction of *R1* alleles transiently arrests cell cycle progression in the late G_1_ phase by affecting the assembly of Cdc45 into the pre-RC and thus delays the activation of the pre-RC at the origins of DNA replication (Chabes and Stillman, 2007). Moreover, inhibition of RNR activity with hydroxyurea impedes DNA replication and cell cycle progression by inducing a slow DNA replication mode with a 25-fold reduction of the initiation rate and a 10-fold reduction of the elongation rate, thus extending the time required for S-phase completion by at least 8 h in budding yeast (Poli *et al.*, 2012).

Although extensive effort has been invested in studying the effects of disrupted RNR activity on DNA replication and cell cycle progression in yeast and human cells, this phenomenon has been unclear in higher plants. Although *rnr* mutants of both large and small subunits have been described in Arabidopsis (large subunit, *cls8*; small subunits, *tso2*, *rnr2a*, *and rnr2b*) (Wang and Liu, 2006; Garton *et al.*, 2007) and *Oryza sativa* (large subunit, *v3*; small subunit, *st1*) (Yoo *et al.*, 2009), the cited studies were mainly concerned with their effects on chloroplast biogenesis. All *rnr* mutants characterized in higher plants have been found to exhibit decreased dNTP pools and reduced chloroplast biogenesis (Wang and Liu, 2006; Garton *et al.*, 2007; Yoo *et al.*, 2009), which suggests a strong correlation between cellular dNTP concentrations and chloroplast biogenesis. In Arabidopsis, the *cls8* mutant and RNAi lines with a disrupted large subunit gene produce bleached leaves and siliques (Garton *et al.*, 2007), while rice *v3* and *st1* mutants develop striped leaves in a growth stage-dependent manner (Yoo *et al.*, 2009). In addition to producing bleached leaves and siliques, *tso2-1* and *tso2-1 rnr2a-1* exhibit obvious developmental defects, including callus-like floral organs, fasciated shoot apical meristems, and defects in cell cycle progression (Wang and Liu, 2006). However, although interesting, these results do not explain how reduced dNTP pools affect cell cycle progression.

In this study, we identified a RNR large subunit mutant, *sistl1*, which produced defective RNRL protein (SiSTL1) and exhibited growth retardation and striped leaf phenotype. Cross sections and microscopic observations of the striped leaves revealed that reduced chloroplast biogenesis and asymmetric leaf cell development occurred in *sistl1*. Yeast two-hybrid (Y2H) analysis revealed that Gly_737_ to Glu substitution of the SiSTL1 protein weakened its interaction with the RNR small subunit. RNA-seq analysis suggested that genes involved in DNA replication and cell cycle progression were repressed in *sistl1*.

## Materials and methods

### Plant materials and growth conditions

A *sistl1* mutant was isolated from ethylmethane sulfonate (EMS)-treated *S. italica* ‘Yugu1’ (foxtail millet). After isolation, the mutant was backcrossed with Yugu1, and recessive derivatives from the backcrosses were used in subsequent experiments. All plants were grown in experimental fields in Beijing or Hainan, China, during the foxtail millet growth season.

### Germination trials

For germination trials, seeds of Yugu1 and *sistl1* were placed on two layers of wet filter paper. Root and shoot lengths and numbers of germinated seeds were determined every 24 h. In addition, germinated seeds of *sistl1* and Yugu1 were photographed at 24, 36, and 48 h after placement on wet filter paper. Each germination trial involved 100 seeds per container, with three replicates. For root and shoot length measurements, 15 Yugu1 and *sistl1* seedlings were used each (each seedling as a biological replicate).

### Leaf structure and chloroplast ultrastructural observation

Fragments of fifth leaves of Yugu1 and *sistl1* were observed under optical (DMLB, Leica, Wetzlar, Hessen, Germany) and confocal (LSM700, Zeiss, Oberkochen, Baden-Wurttemberg, Germany) microscopes. Leaf fragments were gradiently dehydrated with 75%, 85%, 95%, and 100% ethanol and then rendered transparent with 1:1 ethanol: xylene followed by 100% xylene. After washing with 100%, 85%, 65%, 30% ethanol and water, the fragments were stained with I_2_–KI solution. To generate resin-embedded sections, the leaf tissues were fixed with 2.5% glutaraldehyde, washed three times with 0.2 M phosphate buffer, fixed in 1% osmium tetroxide for 1 h, stained with uranyl acetate and subjected to dehydration using an ethanol gradient. After dehydration, the leaf tissues were embedded into resin. The resin blocks were sectioned with a glass blade, and the slices were observed under a transmission electron microscope (JEM 1230, JEOL, Tokyo, Japan). Density curves of bundle sheath cells (BSCs)/mesophyll cells (MCs) containing zero to six chloroplasts were constructed with resin sections of the fifth leaves of Yugu1 and *sistl1*. For each density curve, we counted the number of chloroplasts in four vascular bundles which containing >20 BSCs and >70 MCs.

### Map-based cloning and whole-genome resequencing

For map-based cloning, we used 891 recessive individuals of an F_2_ population generated from a cross between *sistl1* and *S. italica* ‘SSR41’. A total of 132 markers were used to localize the *SiSTL1* gene to a 91-kb interval on chromosome 4. Details of simple sequence repeat (SSR) markers CAAS 4023, CAAS 4019, and CAAS 4033 are given in Zhang *et al.* (2014), and primer sequences of insertion–deletion (InDel) markers and cleaved amplified polymorphic sequence (CAPS) markers are described in Supplementary Table S1 at *JXB* online.

For the whole-genome resequencing, two DNA pools were constructed with 30 Yugu1 and *sistl1* individuals each. Raw data were obtained using the Illumina HiSeq 2500 platform and uploaded with EMBL-EBI in the European Nucleotide Archive database under the accession number PRJEB27720. After quality control, clean data were generated as described in the ‘RNA-seq analysis’ section. Picard tools v1.41 (http://broadinstitute.github.io/picard/) and samtools v0.1.18 (http://www.htslib.org/) were used to sort, remove duplicated reads from and merge the BAM alignment results. For single nucleotide polymorphism (SNP) calling, reads of *sistl1* were input into GATK2 software with *S. italica v2.2* as the reference genome. Raw vcf files were filtered with the GATK standard filter method and other parameters (cluster Window Size: 10; MQ0≥4 and (MQ0/(1.0×DP))>0.1; QUAL<10; QUAL<30.0 or QD<5.0 or HRun>5), and only SNPs with distance >5 were retained. SNPs present within the 91-kb interval of *sistl1* were then filtered by the same SNP calling steps with reads of Yugu1 and other *Setaria* mutants, *SiDWARF3*, and *Loose Panicle1* (Fan *et al.*, 2017; Xiang *et al.*, 2017).

### Knock-out of *SiSTL1* homologous gene in rice

Sequences of RNRL proteins from Arabidopsis (AtRNRL, *At2G21790*), *O. sativa* (OsRNRL1, *LOC_Os06g07210.1* and OsRNRL2, *LOC_Os02g56100*), *Zea mays* (*GRMZM2G304362* and *GRMZM2G340527*), *Sorghum bicolor* (*Sobic.010G054600* and *Sobic.004G336100*), and *Setaria italica* (SiSTL1, *Seita.4G058800*; SiSTL1-2, *Seita.5G216600*; and SiSTL1-3, *Seita.1G356500*) were downloaded from Phytozome v12. A phylogenetic analysis was carried out using MEGA 5 software.

For knock-out of the *OsRNRL1* gene (*LOC_Os06g07210*) in rice, pYLCRISPR/Cas9-MH vectors were constructed as described by Ma *et al.* (2015) and subsequently transferred into rice cultivar Kitaki (*japonica*). To verify whether the transgenic plants contained the pYLCRISPR/Cas9-MH vector, PCR was performed with a primer pair specific for *Cas9* gene amplification (crispr V3 F and R). To verify whether sequence variation occurred, *OsRNRL1* was amplified and sequenced with primer pair OsCas9-C F and R. To verify what kinds of variation occurred in the striped T_0_ transgenic plants, OsCas9-C PCR products from *OsC1-2*, *OsC1-8*, *OsC1-4*, and *OsC1-8* were cloned using the pEASYTM-Blunt Zero Cloning Kit (CB501-02, Transgen Biotech, Beijing, China) and sequenced with monoclone. To verify whether variations occurred in the descendants of the aforementioned transgenic plants, OsCas9-C PCR products of these transgenic descendants were sequenced and the results were analysed using DSDecodeM (http://skl.scau.edu.cn/dsdecode/). Sequence variation of *OsC1-8 T1-1* was too complicated to be resolved by DSDecodeM, and thus its OsCas9-C PCR product was cloned using the pEASY-Blunt Zero Cloning Kit and sequenced with monoclone. Primers used for vector construction and transgenic plants verification are listed in Supplementary Tables S2 and S3.

### Quantitative real-time RT-PCR and subcellular localization

To investigate the expression patterns of *SiSTL1*, *SiSTL1-2*, and *SiSTL1-3* along leaf developmental gradients, four leaf fractions of striped fourth leaves of *sistl1* and normal fourth leaves of Yugu1 were extracted as described by Li *et al.* (2010). After extraction of total mRNA with a Pure Link RNA mini kit (cat. no. 12183018; Invitrogen, Carlsbad, CA, USA), cDNAs were obtained using a PrimeScript first-strand cDNA synthesis kit (cat. no. 6210A; TakaRa, Otsu Shiga, Japan). Quantitative real-time RT-PCR (qRT-PCR) was performed using Fast Start Universal SYBR Green Master Mix (ROX) (cat. no. 04913914001, Roche, Mannheim, Germany) on an Applied Biosystems 7300 Analyser (Applied Biosystems, Foster City, CA, USA). Relative gene expression levels were calculated with the 2^−Δ*C*t^ method. *cullin* (*Seita.3G037700*), described in Martins *et al.* (2016), was used as the reference gene. Relative expression levels of *E2F1* and *E2F2* in the leaf base of *sistl1* and Yugu1 were also obtained in the same way as described above. For qRT-PCR to study the expression changes in *sistl1* and Yugu1 of 11 genes considered to be involved in cell cycle progression, the relative expression of these genes were calculated with *cullin* as reference gene and the 2^−ΔΔ*C*t^ calculation method, as described by Winer *et al.* (1999). For the qRT-PCR conducted with rice transgenic plants, RNA was extracted from the basal region of the seventh leaf, and relative expression of the genes was determined in the same way as described above but using 2^−ΔCt^ calculation method and *Actin* as reference gene, as described by Wang *et al.* (2017). Primers used for qRT-PCR are listed in Supplementary Table S4.

For determination of subcellular localization, *SiSTL1* was fused to a p16318:GFP vector, which was then transferred into protoplasts isolated from fresh leaves of 7-day-old foxtail millet seedlings by a polyethylene glycol-mediated method (Kim *et al.*, 2015*b*).

### Y2H analysis

A Y2H assay was conducted using a Matchmaker Gold Yeast Two-Hybrid system (cat. no. 630489; Clontech, Mountain View, CA, USA). The wild-type SiSTL1 (Gly_737_) allele and the mutant SiSTL1 (Glu_737_) allele were separately fused to AD vectors, while the RNR small subunit gene (*SiRNRS*, *Seita.4G114600*) was fused to a BD vector. The fused AD and BD vectors were then co-transferred into Gold *S. cerevisiae*. The transformed yeast strains were tested for viability on SD/−Ade/−His/−Leu/−Trp/X-α-gal plates.

### RNA-seq analysis

For RNA-seq libraries, we used mRNA of the basal region from the striped seventh leaves of *sistl1*, a leaf zone that was wrapped in the sixth leaf sheath and 1 cm above the leaf seven ligule, which was supposed to be undergoing active cell division. The corresponding leaf region of Yugu1 was used as a control. A total of six cDNA libraries (three of *sistl1* and three of Yugu1) were sequenced with the Illumina NovaSeq 6000 system, and 150-bp paired-end reads were generated. Clean data were obtained by removing reads containing adapters, reads containing ploy-N and low-quality reads from the raw data. Q20, Q30, GC-content and the sequence duplication level of the clean data were calculated for quality control. High-quality clean reads were then mapped to the reference genome (*Setaria italica v2.2*) using Hisat2 tools (Kim *et al.*, 2015*a*), and only reads with a perfect match or one mismatch were counted. Quantification of gene expression abundances was estimated by reads per kilobase of transcript per million fragments mapped (RPKM). For differential expression analysis, clean reads of *sistl1* and Yugu1 were analysed with the DESeq R package (1.10.1) (http://www.bioconductor.org/packages/release/bioc/html/DESeq.html). Genes with adjusted |log_2_RPKM_*sistl1*/Yugu1_|>0.5 and *P v*alue<0.01 found by DESeq were assigned as differentially expressed. Gene Ontology (GO) enrichment analysis of the differentially expressed genes (DEGs) was implemented by the GOseq R packages described by Young *et al* (2010). To validate the Illumina data, relative expression of 27 genes was investigated in *sistl1* and Yugu1 by qRT-PCR. A high correlation (*R*^2^=0.95) was found between the RNA-seq and qRT-PCR data (see Supplementary Table S5). Raw data were uploaded with EMBL-EBI into the European Nucleotide Archive database under the accession numbers PRJEB25717 and PRJEB26878.

### Flow cytometry

For flow cytometry, approximately 30 three-day-old first leaves were cut into pieces in nuclear extraction buffer as described by Lin *et al.* (2012). The extract was stained with 2.5 mg ml^−1^ 4′,6-diamidino-2-phenylindole for 5–10 min and then analysed on a MoFlo XDP cytometer (Beckman Coulter, CA, USA). A total of 8000 nuclei were counted per trial, with three repeats.

## Results

### *sistl1* exhibits delayed growth and a striped leaf phenotype

**Fig. 1.**
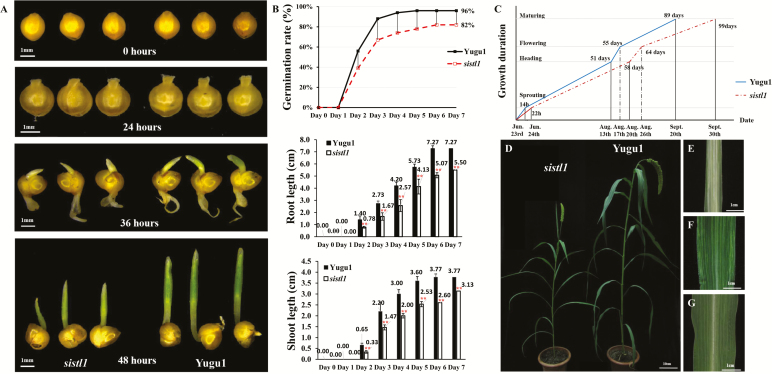
Striped-leaf phenotypes and delayed germination and growth of *sistl1*. (A) Germination status of *sistl1* (left) and Yugu1 (right) at 0, 24, 36, and 48 h. (B) Germination rate, root length, and shoot length of *sistl1* and Yugu1. Asterisks indicate a significant difference between root length and shoot length of *sistl1* and Yugu1; error bars, ±SD (*n*=15 seedlings), Student’s *t*-test, *P*<0.01. (C) Growth duration of *sistl1* and Yugu1 indicating dates of different developmental stages. (D) Morphology of *sistl1* and Yugu1 grown in Beijing (40°N, 116°E; high temperatures and long days). (E) Fifth leaves of *sistl1* grown in Hainan in the winter (19°N, 110°E; low temperatures and short days). (F, G) Fifth leaves of *sistl1* (F) and Yugu1 (G) grown in Beijing in the summer.

Compared with wild-type Yugu1, *sistl1* displayed obvious developmental retardation. According to our germination trials, the germination rate of *sistl1* (82%) was much lower than that of Yugu1 (96%), and root and shoot lengths of *sistl1* were significantly shorter than those of Yugu1 at each observation time point (Fig. 1B). In addition, the germination time of *sistl1* was delayed by 8 h. The radicles of 50% of wild-type Yugu1 seeds penetrated the episperm within 14 h after placement on wet filter paper, whereas most *sistl1* radicles did not emerge for 22 h (Fig. 1A). Furthermore, a comparative examination of the overall growth period of *sistl1* and Yugu1 revealed that developmental stages of *sistl1* plants were delayed to varying degrees: sprouting time by 8 h, heading date by 7 days, and flowering and maturation dates by 9 and 10 days, respectively (Fig. 1C). These observations indicate that *sistl1* experienced developmental retardation.

Another characteristic of *sistl1* was the production of striped leaves in a growth-stage- and environment-dependent manner. For example, in the summer in Beijing (40°N, 116°E) under high-temperature and long-day field conditions, *sistl1* exhibited the normal green leaf phenotype up to the third-leaf stage, and then produced striped fourth and fifth leaves (Fig. 1F). In contrast, in the winter in tropical Hainan (19°N, 110°E) under low-temperature and short-day conditions, *sistl1* produced striped second and third leaves, and the striped area was much larger (Fig. 1E). Leaves generated after the late shooting stage, such as ninth and later leaves, were much less prone to being striped. In favorable field conditions, striped leaves were sometimes nearly absent after the late shooting stage.

### *sistl1* had an abnormal leaf vein arrangement and reduced chloroplast biogenesis

**Fig. 2.**
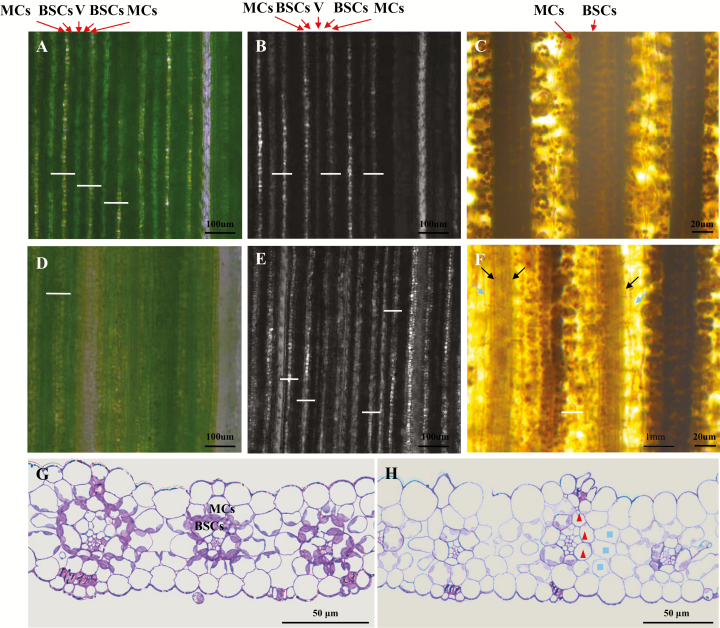
Abnormal leaf vein arrangement and asymmetric cell development of *sistl1*. (A, B) Fifth-leaf fragments of Yugu1 observed by optical (A) and confocal (B) microscopy. Arrows indicate the locations of mesophyll cells (MCs) and bundle sheath cells (BSCs) in one vascular bundle. White bars show the distance between two adjacent veins. (D, E) Fifth-leaf fragments of *sistl1* observed by optical (D) and confocal (E) microscopy. (C, F) I_2_–KI-stained fifth-leaf fragments of Yugu1 (C) and *sistl1* (F). Dark and light arrows indicate unstained BSCs and MCs. (G, H) Resin-embedded sections of Yugu1 (G) and *sistl1* (H) fifth-leaf fragments. Triangles and squares denote abnormal BSCs and MCs in one vascular bundle.

In C_4_ plants such as foxtail millet, vascular bundles, which are surrounded by a layer of bundle sheath cells (BSCs) plus another layer of mesophyll cells (MCs), are arranged in a MC–BSC–V–BSC–MC pattern, an organization referred to as a Kranz structure. Because BSCs are packed with large chloroplasts and tightly organized, they appear deep green under an optical microscope (Fig. 2A). Mesophyll cells have fewer, smaller chloroplasts and are loosely arranged. When MCs are observed under an optical microscope, light spots can be seen as light transmitted from MCs to the eyepiece (Fig. 2A). Vascular bundles are surrounded by BSCs and MCs and thus appear pale green. Similarly, BSCs are very dark under a confocal microscope, MCs are light-spotted and vascular bundles are gray because of their different transmittances (Fig. 2B).

To characterize striped *sistl1* leaves in more detail, fragments of the fifth leaves of Yugu1 and *sistl1* were observed under optical and confocal microscopes (Fig. 2A, B, D, E). Yugu1 leaves were found to possess a well-organized MC–BSC–V–BSC–MC pattern (Fig. 2A, B), with a uniform distance between adjacent veins (white bars in Fig. 2A, B). In *sistl1* leaf fragments, in contrast, the MC–BSC–V–BSC–MC pattern was disrupted, and the distance between adjacent veins decreased irregularly (Fig. 2D, E). After staining with I_2_–KI, many unstained MCs and BSCs were observed in *sistl1* (Fig. 2F), which suggests that these cells lacked chloroplasts and thus could not accumulate starch. Observations of resin-embedded sections of *sistl1* striped leaf fragments also identified some MCs and BSCs with no chloroplasts; consequently, they were not stained by toluidine blue and appeared as ‘empty’ cells (triangles and squares in Fig. 2H). In addition, some veins of *sistl1* were only half normal in structure, indicating the possibility of asymmetric cell development along the vein axis.

**Fig. 3.**
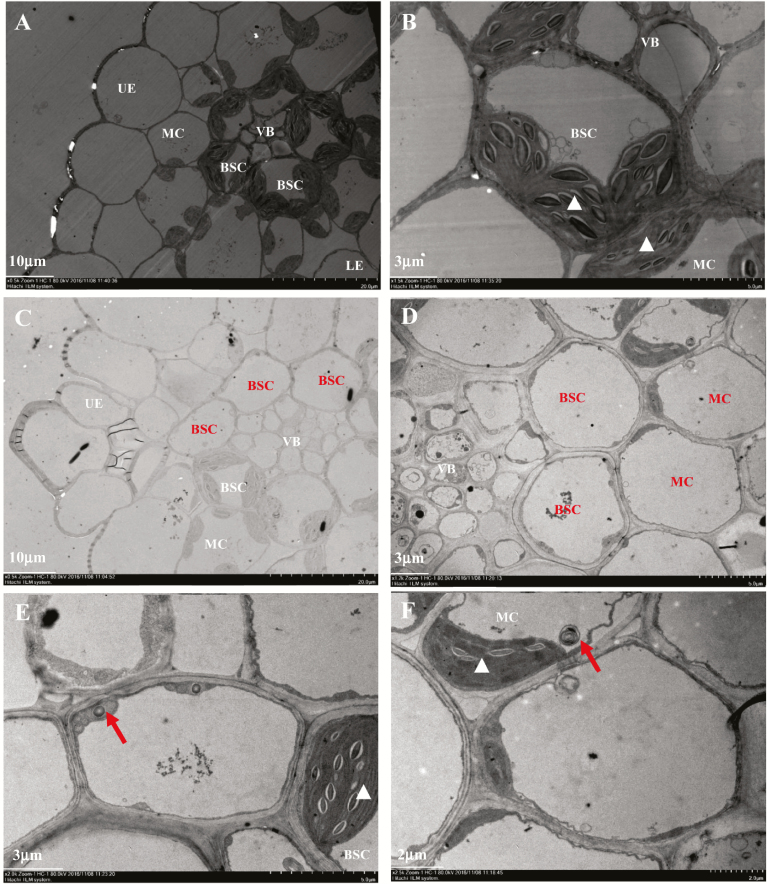
Reduced chloroplast biogenesis in *sistl1*. (A, B) Ultrastructure of Yugu1 fifth-leaf fragments observed by transmission electron microscopy (TEM) under ×0.5K (A) and ×1.5K (B) magnification. (C–F) Ultrastructure of fifth-leaf *sistl1* fragments observed by TEM under ×0.5K (C), ×1.2K (D), ×2.0K (E) and ×2.5K (F) magnification. Normal cells in Yugu1 and *sistl1* are labeled with white letters, with white triangles indicating normally developed chloroplasts in BSCs and MCs. Unusual empty cells in *sistl1* are labeled with dark letters. Dark arrows indicate lysosome- or peroxisome-like organelles.

Ultrastructural observation revealed that the chloroplasts of *sistl1* were indistinguishable from those of Yugu1 (white triangles in Fig. 3B, E, F), but the number of chloroplasts per BSC and MC was reduced dramatically (Figs 2G, H, 3A, C). According to our observation, most BSCs and MCs of Yugu1 contained two to four chloroplasts, whereas in *sistl1*, BSCs and MCs with no chloroplasts accounted for the largest proportion of cells (see Supplementary Fig. S1). Furthermore, we found that cells lacking chloroplasts produced many lysosome- and peroxisome-like organelles (Fig. 3E, F). All of these observations indicate that *sistl1* had reduced chloroplast biogenesis and exhibited asymmetric leaf cell development.

### Map-based cloning of the *SiSTL1* gene

**Fig. 4.**
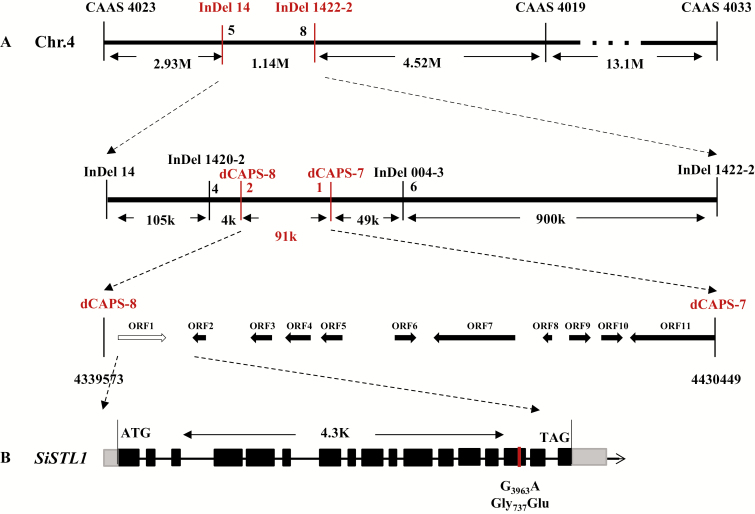
Map-based cloning of the *SiSTL1* gene. (A) Map-based cloning of the *SiSTL1* gene. Thick arrows indicate 11 candidate open reading frames (ORF) within the 91-kb interval. The white arrow indicates the mutant gene. (B) Schematic diagram of *SiSTL1*. Black boxes indicate the exons and black lines indicate introns of *SiSTL1*.

Genetic mapping of the *SiSTL1* gene was performed using F_2_ individuals generated from a cross between the *sistl1* mutant and foxtail millet cultivar SSR41. Using PCR-based markers, the *SiSTL1* locus was initially mapped to an 8.6-Mb region between two SSR markers, CAAS 4023 and CAAS 4019, on chromosome 4 (Fig. 4). To generate a fine mapping, InDel and CAPS markers were developed by comparing the genomic sequences of Yugu1 and SSR41. The *SiSTL1* locus was finally narrowed to a 91-kb interval between CAPS-8 (4339573 on chromosome 4) and CAPS-7 (4430449 on chromosome 4). In this region, three SNPs were identified by whole-genome resequencing of Yugu1 and *sistl1* (see Supplementary Table S6) using Illumina next-generation sequencing technology. The first SNP, in the 15th exon of *Seita.4G058800* (Chr4: 4347392), was a non-synonymous G_3963_A mutation leading to a missense mutation (Gly_737_ to Glu) in the encoded RNR large subunit protein (SiSTL1). The second SNP occurred in an intergenic region (Chr4: 4412036). According to the genome annotation of *Setaria italica v2.2*, the region 5000 bp upstream and downstream of this position does not contain a gene or ncRNA. The third SNP was located in the first exon of *Seita.4G059700*. It was a synonymous mutation also found in the *Setaria* mutants *SiDWARF3* and *Loose Panicle1* (Fan *et al.*, 2017; Xiang *et al.*, 2017). As described in Fan *et al.* (2017) and Xiang *et al.* (2017), *SiDWARF3* and *Loose Panicle1* both have dwarf and loose panicle phenotypes, but neither demonstrated the growth retardation or striped leaf phenotype that was seen for *sistl1*. The causal genes of the *SiDWARF3* and *Loose Panicle1* mutations were located in chromosomes 8 and 2, respectively. We therefore confirmed that the third SNP in the *SiSTL1* locus was a background SNP. Notably, like *sistl1*, mutants of the RNR large subunit gene in Arabidopsis (*cls8*) and rice (*v3*) exhibit bleached and striped leaves (Garton *et al.*, 2007; Yoo *et al*, 2009). We therefore confirmed that the non-synonymous SNP occurring in the 15th exon of *Seita.4G058800* was responsible for the observed mutant phenotypes of *sistl1*.

### Functional verification of *SiSTL1* in rice

**Fig. 5.**
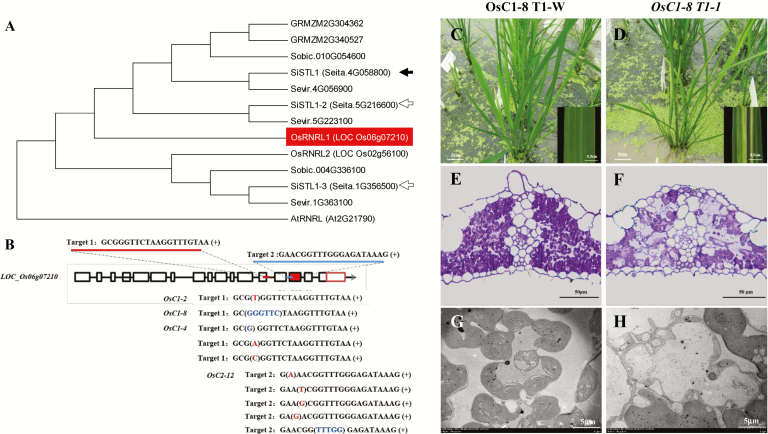
Knock-out of *SiSTL1* homologous gene in rice. (A) Phylogenetic tree of RNRLs in Arabidopsis, *Oryza sativa*, *Zea mays*, *Sorghum bicolor*, and *Setaria italica* based on protein sequences. Black arrow indicate the causal gene for *sistl1*. White arrows indicate two other genes encoding SiSTL1 homologues in *Setaria* (*SiSTL1-2* and *SiSTL1-3*). The homolog of *SiSTL1* in rice (*OsRNRL1, LOC Os06g07210*) is highlighted by shading. (B) Position of two CRISPR targets in *OsRNRL1* and sequence variations in the striped transgenic T_0_ plants, *OsC1-2*, *OsC1-4*, *OsC1-8*, *OsC2-12*, and three other chimeric plants. (C, D) T_1_ plants of *OsC1-8* grown in a paddy field. *OsC1-8 T1-W*, normal green individual; *OsC1-8 T1-1*, striped-leaf individual. (E, F) Resin sections of *OsC1-8 T1-W* (E) and striped *OsC1-8 T1-1* (F) leaf fragments. (G, H) Ultrastructures of *OsC1-8 T1-W* (G) and striped *OsC1-8 T1-1* (H) leaf fragments.

Because a transfection system has not been fully established for *Setaria*, functional verification of *Seita.4G058800* was carried out in rice. According to our phylogenetic analysis, *LOC_Os06g07210* is orthologous to *SiSTL1* in rice (Fig. 5A). We therefore constructed two pYLCRISPR/Cas9-MH vectors respectively targeting exons 13 and 15 (Fig. 5B) of *LOC_Os06g07210*. Of the subsequently generated positive transgenic T_0_ plants, one homozygous mutant (*OsC1-2*) and three heterozygous mutants (*OsC1-4*, *OsC1-8*, and *OsC2-12*) of *LOC_Os06g07210* were obtained and exhibited striped leaves just like the *sistl1* and rice *v3* mutants (Yoo *et al*, 2009) (see Supplementary Fig. S2A). *OsC1-2* was homozygous for a single T-nucleotide insertion in exon 13 (Fig. 5B) that was responsible for the loss of 168 amino acids from the C-terminus of the RNRL protein. We were unable to obtain T_1_ plants of this mutant, however, as all of them were albino and died at the early seedling stage, suggesting that the frame-shift mutation that occurred in this locus is lethal in homozygotes. We therefore observed the T_1_ lines of the heterozygous mutants. *OsC1-8* had a 6-bp deletion in exon 13 (Fig. 5B), which resulted in the loss of two amino acids from the protein. It also produced albino T_1_ seedlings, and we only obtained one striped T_1_ plant (*OsC1-8 T1-1*) (Fig. 5D). Resin-section and ultrastructural assays of its leaf fragments showed that some of the mesophyll cells lacked chloroplasts (Fig. 5F, H). *OsC1-4* had a G deletion in exon 13 (Fig. 5B), and three striped T_1_ plants were obtained among its T_1_ descendants (Supplementary Fig. S2B). A variation of *OsC2-12* was an A insertion in exon 15 (Fig. 5B), and we also obtained three T_1_ plants that displayed pronounced stripe-leaf phenotype (Supplementary Fig. S2C). What is more, although in a small proportion, two striped descendants were also obtained among the descendants of *OsC1-8 T1-1* (Supplementary Fig. S2D). We thus confirmed that the striped-leaf phenotype of all these transgenic plants was heritable. To study whether the striped leaf phenotype was tightly linked to the CRISPR-induced mutation, the targeted gene of the striped T_1_ and T_2_ descendants was sequenced. The result showed all of them contained variations in the targeted gene. *OsC1-8 T1-1* was a chimeric mutant that contained the wild-type gene sequence, a 6-bp deletion in exon 13 that was inherited from *OsC1-8*, and a G deletion in exon 13 (Supplementary Fig. S3B). Genotypes of all three striped T_1_ descendants of *OsC1-4* were the same as each other. As shown in Supplementary Fig. S3C, they were also chimeric mutants, containing the wild-type sequences, an A insertion in exon 13, and a T insertion in exon 13. Striped T_1_ descendants of *OsC2-12* were heterozygous mutants. As showed in Supplementary Fig. S3D, variation of *OsC2-12 T1-1* was the same as that of its parent *OsC2-12*; *OsC2-12 T1-2* contained a C insertion in exon 15. *OsC2-12 T1-3* had an A deletion in exon 15. As new variations were found in the striped T_1_ and T_2_ descendants, PCR was carried out with primers specific for *Cas9* gene amplification, which showed that all the striped T_1_ and T_2_ descendants still contained the pYLCRISPR/Cas9-MH vector (Supplementary Fig. S4), suggesting that new variations occurring in these descendants might result from secondary editing by Cas9 in the plants. Taking all these results together, we tentatively concluded that mutations of *LOC_Os06g07210* result in a striped-leaf phenotype and the striped leaf phenotype of *sistl1* was caused by the G_3963_A base substitution in the *SiSTL1* gene.

### *SiSTL1* is preferentially expressed in younger leaf tissues.

Phylogenetic analysis revealed the presence of three genes encoding RNRL in *Setaria*: *Seita.4G058800* (*SiSTL1*), *Seita.5G216600* (*SiSTL1-2*), and *Seita.1G356500* (*SiSTL1-3*) (black and white arrows in Fig. 5A). To investigate the expression pattern of these genes along leaf developmental gradients, four leaf fractions of the striped fourth leaves of *sistl1* and normal fourth leaves of Yugu1 were extracted as described in Li *et al.* (2010). The first fraction (LB) was taken from the 1 cm basal region above the fourth leaf ligule. This leaf zone, which was undergoing the most active cell division and was wrapped in the third leaf sheath, had not yet developed any chloroplasts and represented the earliest stage of leaf development (Li *et al.*, 2010). The fourth fraction (LA) was obtained from the 1 cm region beneath the leaf tip. This leaf portion was totally expanded and contained well-developed chloroplasts (Li *et al.*, 2010). The second and third fractions (LMB and LMA) corresponded respectively to the 1 cm regions beneath and above the third leaf ligule. These fractions represented transition stages for proplastid development into chloroplasts (Li *et al.*, 2010).

**Fig. 6.**
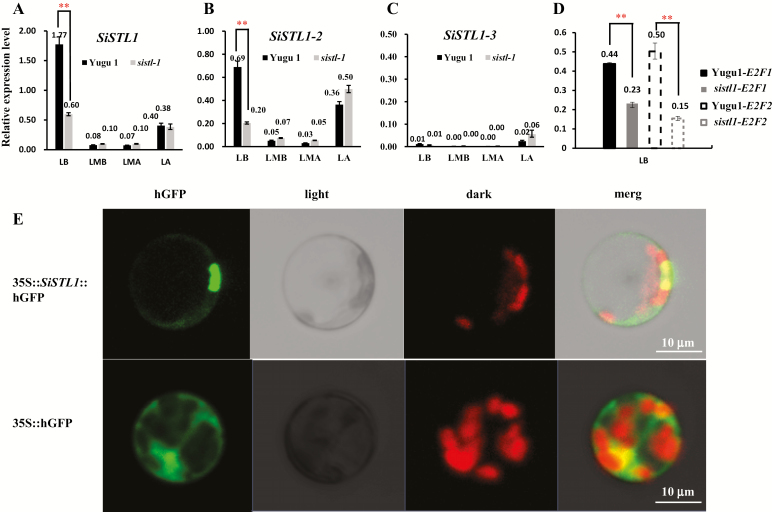
Expression pattern of *SiSTL1* and subcellular location of SiSTL1. (A–C) Expression patterns of *SiSTL1* (A), *SiSTL1-2* (B), and *SiSTL1-3* (C) along leaf developmental gradients in Yugu1 and *sistl1* fourth leaves. LB, basal 1 cm region above the fourth leaf sheath; LMB, 1 cm region beneath the third leaf sheath; LMA, 1 cm region above the third leaf sheath; LA, 1 cm region beneath the leaf tip. Asterisks indicate a significant difference between the relative expression level of genes in Yugu1 and *sistl1* fourth leaf base; error bars, ±SD (*n*=3 replicates), Student’s *t*-test, *P*<0.01. (D) Expression patterns of *E2F1* and *E2F2* in Yugu1 and *sistl1* fourth-leaf bases. (E) Subcellular localization of the SiSTL1 protein in *Setaria* protoplast.

According to our qRT-PCR analysis, *SiSTL1* was preferentially expressed in LB, followed by LA (Fig. 6A). Fewer transcripts were detected in LMB and LMA fractions. These results suggest that active RNR activity is required during early leaf cell division. The expression pattern of *SiSTL1-2* was the same as that of *SiSTL1*, but the abundance was much lower (Fig. 6B). Virtually no transcripts of *SiSTL1-3* were detected in any of the leaf tissues (Fig. 6C), indicating that *SiSTL1-3* may be a pseudogene or have functions in other tissues. Notably, compared with Yugu1, relative expression levels of *SiSTL1* as well as its upstream regulators, *E2F1* and *E2F2*, were greatly reduced in the LB fraction of *sistl1* (Fig. 6D), indicating that a feedback mechanism involving the upstream genes *E2F1*, *E2F2*, and *SiSTL1* may operate in *sistl1*.

In a SiSTL1 subcellular localization experiment, hGFP signals were detected in both the nucleus and cytoplasm (Fig. 6E). This observation is consistent with the results of Lincker *et al.* (2004), who reported that RNRL is primarily present in the cytoplasm and can be transferred to the nucleus when RNR activity is needed.

### Gly to Glu substitution in the C-terminus of SiSTL1 weakens its interaction with the RNR small subunit

**Fig. 7.**
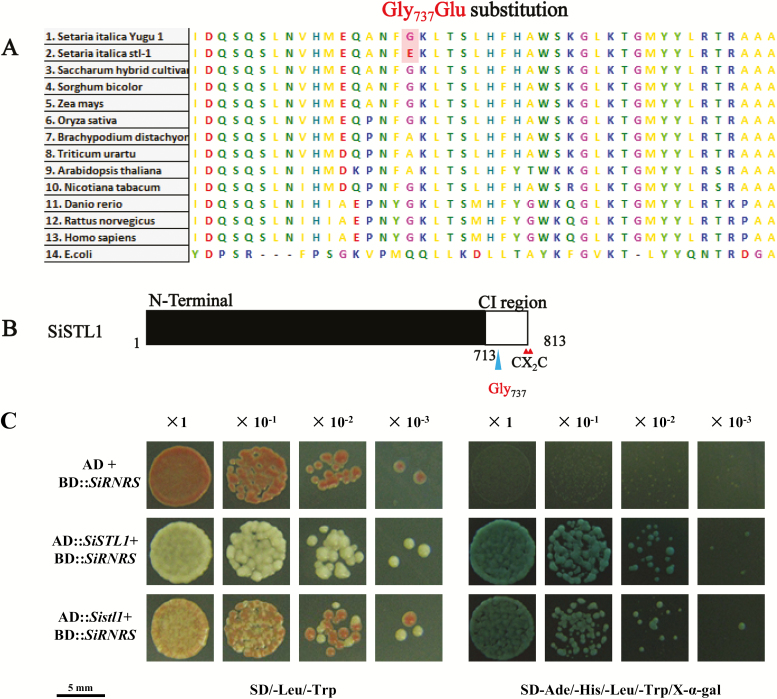
Sequence analysis of SiSTL1 and results of a yeast two-hybrid assay of SiSTL1 and SiRNRS. (A) Comparison of aligned sequences of SiSTL1 and homologous proteins from other species. (B) Schematic diagram of SiSTL1. Small triangles indicate the positions of the CX_2_C motif of SiSTL1. The last ~100 amino acids before the CX_2_C motif are designated as the C-terminal insertion (CI) region. The large triangle indicates the position of Gly_737_ in the CI region of SiSTL1. (C) Yeast two-hybrid analysis of SiSTL1 and SiRNRS. Dilutions are shown at the top (×10^−1^, yeast diluted 10 times; ×10^−2^, yeast diluted 100 times; ×10^−3^, yeast diluted 1000 times).

Sequence analysis indicated that Gly_737_ is not a highly conserved residue. Among 100 eukaryotic RNRL proteins, 78% have Gly in the homologous location (Fig. 7A). Ala was present in 9% of such proteins, with a Lys or Ser in the remainder. Both Gly and Ala are uncharged amino acids, with –H or –CH_3_ as the side chain, respectively. In contrast, Glu is an acidic amino acid that has a negatively charged –(CH_2_)_2_–COO^−^ side chain. The Gly_737_Glu substitution thus changed the charge properties of this part of the protein.

The CI region, which comprises approximately the last 100 amino acids before the CX_2_C motif at the RNRL C-terminus, is important for optimal RNRL activity (Zhang *et al.*, 2007). An *rnr1 rnr3* double mutant of *Saccharomyces cerevisiae* transformed with *rnr1* mutant alleles lacking the CI region grew more slowly than the wild-type (Zhang *et al.*, 2007). We thus speculated that the Gly_737_Glu substitution in the SiSTL1 CI region (Fig. 7B) would affect the function of this protein. To test this hypothesis, Y2H tests were conducted. Wild-type SiSTL1 (Gly_737_) and mutant SiSTL1 (Glu_737_) alleles were fused to AD vectors, and the RNR small subunit (SiRNRS, *Seita.4G114600*) was fused to a BD vector. Y2H Gold yeast cells transformed with the Glu_737_ SiSTL1 allele were visible on SD/−Ade/−His/−Leu/−Trp/X-α-gal medium (Fig. 7C). However, compared with yeast transformed with the wild-type Gly_737_ SiSTL1 allele, they grew slowly, with blue substrate appearing approximately 6 h later. Similar results were obtained from Y2H tests using Gly_737_ and Glu_737_ SiSTL1 alleles respectively fused to BD vectors and SiRNRS fused to an AD vector. These consistent results indicate that the Gly_737_ to Glu substitution in the C-terminus of SiSTL1 does not block the interaction of this protein with the small subunit, but weakens its optimal functioning.

### DNA replication activities are affected in *sistl1*

**Fig. 8.**
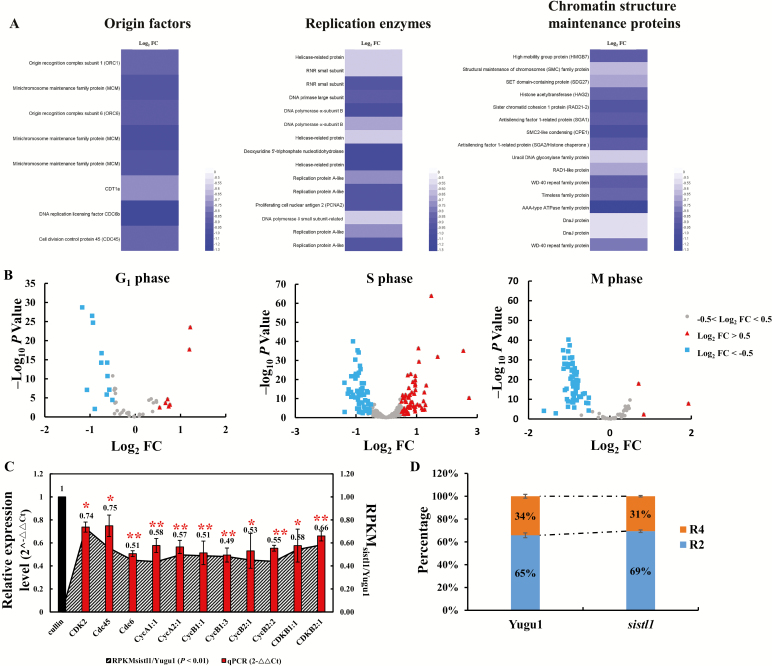
RNA-seq and flow cytometric analysis of Yugu1 and *sistl1*. (A) Heatmaps of genes encoding DNA replication origin factors, replication enzymes or chromosome structural maintenance proteins that significantly reduced gene expression abundances in *sistl1* (log_2_FC<−0.5 and *P*<0.01, log_2_FC=log_2_RPKM_*sistl1*/Yugu1_. RPKM, reads per kilobase of transcript per million fragments mapped). (B) Expression changes of 49, 324 and 107 genes that were preferentially expressed during G_1_, S, and M phase, respectively, between *sistl1* and Yugu1 within the RNA-seq analysis. The *x*-axis is log_2_FC, indicating log_2_RPKM_*sistl1*/Yugu1_ of the discussed genes, and the *y*-axis is −log_10_*P*. The triangles indicate genes where log_2_FC>0.5 and −log_10_*P*>2, which means that the expression level of these genes in *sistl1* was >1.41 times higher than that in Yugu1. The squares indicate genes where log_2_FC<−0.5 and −log_10_*P*>2, which means that the expression level of these genes in *sistl1* was >1.41 times down-regulated in Yugu1. The gray circles represent genes where −0.5<log_2_FC<0.5, which means that the expression level of these genes in *sistl1* had no significant difference compared with that of in Yugu1. Detailed information, such as gene ID, log_2_FC, *P*-value and preferential expression phase of the genes shown in the figure is listed in Supplementary Table S8. (C) Relative expression levels of 11 representative cell cycle regulatory genes in *sistl1*. Left *y*-axis shows the 2^−ΔΔ*C*t^ values for these genes with a qRT-PCR assay (with *Cullin* as the reference gene). Right *y*-axis shows the RPKM_*sistl1*/Yugu1_ values for these genes extracted from RNA-seq. Asterisks indicate a significant difference between the relative expression level of genes in Yugu1 and *sistl1*; error bars, ±SD (*n*=3 replicates), Student’s *t*-test, **P<0.01; *P<0.05. (D) Results of flow cytometric analysis of 3-day-old first-leaf cells. R4 indicate 4C cells, and R2 indicate 2C cells.

Because inhibition of RNR activity with hydroxyurea slows DNA replication in budding yeast (Poli *et al.*, 2012), we presumed that DNA replication activity in *sistl1* would also be affected. To verify this assumption, we analysed expression abundances of genes involved in replication activities by RNA-seq analysis using the basal zone of the striped seventh leaves of *sistl1* and corresponding leaf region of Yugu1. Expression abundances of genes involved in initiation and activation of replication origins, including *MCMs*, *ORC1*, *ORC6*, *Cdc6*, *Cdt1* and *Cdc45*, and genes encoding multiple replication enzymes and chromatin structure maintenance proteins, including DNA polymerase α- and δ-subunits, DNA primase large subunit, helicase-related proteins and structural maintenance of chromosomes family proteins, were significantly reduced in *sistl1* (Fig. 8A; Supplementary Table S7). These observations strongly suggest that DNA replication activities are impeded in *sistl1*.

### Cell cycle progression is arrested in *sistl1*

To verify whether cell cycle progression is affected in *sistl1*, we further explored the RNA-seq results. Menges *et al.* (2003) identified a set of 1082 cell cycle-regulated genes, among which 129, 669, 20, and 198 had expression peak in the G_1_, S, G_2_, and M phase, respectively, by analysis of gene expression profiles during synchronous cell cycle progression with Arabidopsis cell suspension. We blasted these 1082 genes against the *Setaria italica* genome and found 486 *Setaria* homologs present in our RNA-seq list (Supplementary Table S8). And among these 486 genes, 214 genes exhibited significantly different expression patterns (*P*<0.01, |log_2_RPKM_*sistl1*/Yugu1_|>0.5) between *sistl1* and Yugu1. Among the 214 genes, in the research of Menges *et al.* (2003), 20 had peak expression in the G_1_ phase, 120 in the S phase, 3 in G_2_ phase, and 71 in M phase. Interestingly, *Setaria* homologs of the G_1_- and S-phase-specific expressed genes in the research of Menges *et al.* (2003) were approximately half up-regulated (40% and 50.8%) and half down-regulated (60% and 49.2%) in *sistl1*, whereas *Setaria* homologs of the genes preferentially expressed during the M phase were largely down-regulated in *sistl1* (3 (4.2%) up-regulated and 68 (95.8%) down-regulated; Fig. 8B). Because only a few genes were preferentially expressed during the G_2_ phase, they were not taken into account. We thus speculated that a decrease of cells in the M phase in *sistl1* might be the cause of the reduced expression of genes believed to preferentially express during the M phase. To verify this, we conducted a flow cytometric analysis with 3-day-old first-leaf cells. The results indicated that the percentage of 4C cells in *sistl1* was much lower than that of Yugu1 (Fig. 8D), consistent with the assumption that the number of G_2_/M-phase cells was significantly decreased in *sistl1*.

In addition, we performed GO term enrichment analysis for the 1082 cell cycle regulated genes described by Menges *et al.* (2003) and the 214 *Setaria* homologous genes that were differentially expressed between Yugu1 and *sistl1* using the most current annotations (*Tair 10* for Arabidopsis, and *Setaria italica v2.2* for foxtail millet). As we expected, cell cycle-related GO terms were the most abundant terms. Supplementary Fig. S5A exhibits the top 20 most enriched non-redundant biological process GO terms for the 1082 Arabidopsis cell cycle regulated genes. The enriched GO term containing most abundant genes was ‘response to chemical stimulus’, which was consistent with the result described in Menges *et al.* (2003), where the data were generated with aphidicolin- and sugar starvation-treated MM2d cell suspensions. The remaining enriched GO terms were all cell cycle-related (see Supplementary Fig. S5A). The top 20 most enriched GO terms for the 214 DEGs in *sistl1* were also all cell cycle-related (Supplementary Fig. S5B), with the three most significant terms being ‘regulation of mitotic cell cycle’, ‘regulation of cell cycle phase transition’, and ‘DNA replication initiation’. To further explore how these biological processes were affected in *sistl1*, we investigated how many genes were down-regulated and how many up-regulated in these biological processes. For 15 out 20 terms, the genes were all down-regulated in *sistl1* (Supplementary Fig. S5C). The remaining five terms included only two up-regulated genes (three terms) or one up-regulated gene (two terms). These results are highly consistent with our finding that DNA replication and cell cycle progression were impeded in *sistl1*.

Moreover, qRT-PCR analysis of 11 genes considered to be related to cell cycle regulation, specifically, genes promoting S-phase entry (*CDK2*, *Cdc45*, *Cdc6*, *CycA1:1*, *CycA2:1*, *CycB1:1*, *CycB1:3*, *CycB2:1*, *CycB2:2*, *CDKB1:1*, and *CDKB2:1*) (Stevens and and La Thangue, 2003; Yang *et al*., 1999), revealed that they were significantly down-regulated in *sistl1* (Fig. 8C). We also checked the RPKM values of all these genes in our RNA-seq list. The results were completely consistent. RPKM_*sistl1*/Yugu1_ values of all these genes were <1 (*P*-value<0.01), indicating that the relative expression level of all these genes was significantly reduced in *sistl1*. Taken together, our results suggest that cell cycle progression is arrested in the G_1_/S phase in *sistl1*.

## Discussion

### The phenotype of *sistl1* is comparable to *v3* and *cls8* mutants

Mutants of RNRL have been characterized in both Arabidopsis (*cls8*) and rice (*v3*). *v3* produces chlorotic leaves in a growth stage-dependent and temperature-conditional manner (Yoo *et al.*, 2009). In favorable conditions, *v3* generally exhibits a normal green phenotype up to the third-leaf stage, produces chlorotic leaves at the tillering stage, and then produces nearly normal green leaves after heading. If *v3* is grown at a constant 20 °C, a temperature not optimal for growth, the bleached leaf phenotype is more severe, and striped leaves may appear beginning from the second leaf. The Arabidopsis mutant *cls8-1* produces bleached, crinkled leaves because of reduced chloroplast numbers in leaf cells and asymmetric cell development along the vein axis (Garton *et al.*, 2007).

In this study, we identified a RNRL mutant in *Setaria* (*sistl1*) that exhibits a phenotype comparable to *v3* and *cls8-1* (Fig. 1). As described above, *sistl1* produces striped leaves in the same way as *v3*. Compared with fourth and fifth leaves generated during the shooting stage, the first three leaves of *sistl1* and those produced after the late shooting stage are much less likely to exhibit the striped phenotype. When *sistl1* is grown in poor conditions, the striped leaf phenotype is much more pronounced (Fig. 1E). We also observed an abnormal leaf vein arrangement and irregularly reduced leaf vein distances in *sistl1* that may be due to the same phenomenon causing crinkled leaves and asymmetrical flowers in *cls8-1*, namely, asymmetrical development of cells along the vein axis (Fig. 2). Unlike *cls8-1*, however, *sistl1* exhibited obvious growth retardation throughout the entire growth period (Fig. 1A–C), a behavior consistent with the reduced root growth of *cls8-1* and the delayed plant growth of *AtRNRL*-disrupted RNAi lines (Garton *et al.*, 2007).

*SiSTL1* has two homologs in *Setaria* (Fig. 5A). The identity of protein sequences between SiSTL1 and SiSTL1-2 is 98% and the identity between SiSTL1 and SiSTL1-3 is 88%. In *sistl1*, a G_3963_A mutation in *SiSTL1* caused a striped leaf phenotype regardless of the presence of the other two wild-type *SiSTL1* homologs, *SiSTL1-2* and *SiSTL1-3*, indicating that these proteins are not redundant. The reason might be as follows. First, in the qRT-PCR analysis to investigate the expression pattern of these genes along leaf developmental gradients, virtually no transcripts of *SiSTL1-3* were detected in any leaf tissues (Fig. 6C; Supplementary Fig. S6). We thus think that *SiSTL1-3* might be a pseudogene or functional in other tissues, and is not redundant with *SiSTL1*. Although the expression pattern of *SiSTL1-2* was similar to that of *SiSTL1*, its abundance was much lower (Fig. 6B). Thus, it cannot fully complement the functional defects caused by the G_3963_A mutation of *SiSTL1* in *sistl1*. In addition, relative expression levels of *SiSTL1* as well as its upstream regulators *E2F1* and *E2F2* were greatly reduced in the LB fraction of *sistl1* (Fig. 6A, D), indicating that a positive feedback other than negative feedback mechanism involving the upstream genes of *E2F1*, *E2F2* and *SiSTL1* may operate in *sistl1*. We thus presume that *SiSTL1-2* might not be redundant with *SiSTL1*. However, it remains unclear why SiSTL1 and SiSTL1-2 are not functionally reductant although they share such high identity (98%). In the future, we need to undertake more experiments to draw stronger conclusions.

### The function of RNRL is indispensable for plant growth and survival

No frame-shift mutations of RNRL have previously been identified in higher plants. In a study involving *AtRNRL*-disrupted RNAi lines, a quarter of the T_2_ seedlings had a pronounced *cls8* phenotype, and most failed to develop beyond the four-leaf stage (Garton *et al.*, 2007). Only heterozygotes were able to produce seeds, and homozygotes were unlikely to survive. In the current study, *OsRNRL1* (*LOC_Os06g07210*) was targeted using the Cas9 protein. The T_1_ seedlings of *OsC1-2*, a homozygous frame-shift mutant, were unable to survive to the two-leaf stage. The T_1_ plants we obtained, which produced striped leaves, were descendants of heterozygous and chimeric T_0_ lines and were heterozygous and chimeric mutants (Supplementary Figs S2, S3). Taking all of this together we presume that activity of RNRL is indispensable for plant growth and survival, and transgenic descendants with either homozygous frame-shift mutations of the gene or dramatically decreased gene expression are unable to survive. In fact, heterozygous mutants *OsC1-8*, *OsC1-4* and *OsC2-12* also produced albino descendants. We presumed that these albino seedlings were homozygous frame-shift variations that derived from their heterozygous parents.

### *SiSTL1* functionally corresponds to *OsRNRL1*

In the paper describing the *v3* mutant, Yoo *et al.* (2009) proposed that upon insufficient activity of RNR, plastid DNA synthesis is preferentially arrested to allow nuclear genome replication in developing leaves, enabling continuous plant growth. In other words, chloroplast biogenesis is vulnerable to insufficient activity of RNR. To verify the functionally corresponding relationship of *SiSTL1* and *OsRNRL1*, we selected five genes (*OsRNRL1* and four genes involved in cell cycle progression, corresponding to genes that were down-regulated in *sistl1—OsE2F1*, *OsCDK2*, *OsCycA1:1* and *OsCycB1:1*) to perform qRT-PCR using the RNA of striped and normal T_1_ lines of *OsC1-4*, *OsC2-12*, and striped and normal T_2_ lines of *OsC1-8*. Compared with normal descendants, relative expression of all these genes was down-regulated in the striped descendants (see Supplementary Fig. S7). Thus, we confirmed that indeed *SiSTL1* functionally corresponds to *OsRNRL1*. Taking all of these results together, we tentatively conclude that mutations of *LOC_Os06g07210* result in a striped-leaf phenotype and the striped leaf phenotype of *sistl1* was also caused by the G_3963_A base substitution in the *SiSTL1* gene.

### The CI region is important for the function of the RNR large subunit.

Higher plants with defective RNRL proteins feature reduced chloroplast biogenesis (Garton *et al.*, 2007; Yoo *et al.*, 2009). Notably, both *cls8* and *v3* are missense mutants with only a single amino acid alteration, namely, Gly_718_Ala in *cls8-1* and Gly_291_Ser in *v3*. These residues are conserved in higher plants, but are not considered key residues in the catalytic site (Garton *et al.*, 2007; Yoo *et al.*, 2009). In this study, we identified another missense RNRL mutant, *sistl1* in *Setaria*. The altered Gly_737_ is likewise not designated a key residue in allosteric regulation or any catalytic reaction. Indeed, in contrast to the above-mentioned mutations, Gly_737_ is not even highly conserved in higher plants (Fig. 7A). However the Gly_737_Glu substitution changed the charge of the protein and thus may disrupt the function of the CI region. *sistl1* also exhibits a striped leaf phenotype and obvious growth retardation. In a study of *Saccharomyces cerevisiae*, deletion of the whole CI region has been found to affect the optimal function of the RNR large subunit (Zhang *et al.*, 2007). In the present study, we observed that an amino acid substitution within the CI region also affects the function of SiSTL1. Our findings provide new evidence that the CI region is important to the function of the RNR large subunit.

### Slight defects in SiSTL1 affect chloroplast DNA biosynthesis but not chloroplast development

Both *v3* and *cls8* exhibit reduced chloroplast biogenesis, consistent with our observations for *sistl1*. In this study, we conducted a qRT-PCR analysis of three *SiSTL1* gene homologues during the transition to chloroplast from proplastids during leaf development. Relatively few gene transcripts were detected during this phase (Fig. 6A–C), suggesting that RNRL is not crucial for the development of chloroplasts from proplastids. Ultrastructural observation of *sistl1* revealed that cells in striped areas lacked chloroplasts, whereas cells in green areas produced chloroplasts that were indistinguishable from those of Yugu1 (Fig. 3). No undifferentiated chloroplasts were observed in either leaf area. We thus propose that defects in SiSTL1 affect chloroplast DNA synthesis, but not chloroplast development.

### Activation of the S-phase checkpoint may occur in *sistl1*

In yeast and human cells, inhibition of RNR activity leads to reduced cellular dNTP pools and subsequent DNA replication stress and activation of the S-phase checkpoint, thereby restricting the formation of later replication forks in the S phase, increasing dNTP production, and arresting the cell cycle transition (Giannattasio and Branzei, 2017; Pardo *et al.*, 2017). In this study, many genes involved in DNA replication and cell cycle regulation were down-regulated in *sistl1* (Fig. 8A, C). Given the close causal relationship between disrupted RNR activity and the S-phase checkpoint in yeast and human cells (Giannattasio and Branzei, 2017), we propose that the S-phase checkpoint is also activated by the defective SiSTL1 protein in *sistl1* and plays a role in the reduced expression of genes involved in DNA replication and cell cycle regulation.

Notably, DNA replication stress and activation of the S-phase checkpoint always induced increased RNR activity in previous studies (Pardo *et al.*, 2017). However, in our study, relative expression levels of two *SiSTL1* genes and their regulators, *E2F1* and *E2F2*, were all reduced in *sistl1* (Fig. 6A, B, D). Expression abundance of genes encoding DP proteins, which combine with E2F to generate the functional E2F–DP complex, were also decreased in *sistl1* (see Supplementary Table S7). Because ATR–CHK1 up-regulates E2F1 and thus increases RNR activity in human cells (Giannattasio and Branzei, 2017), we believe that other mechanisms exist in *sistl1* to down-regulate the E2F transcription factors when cells encounter DNA replication stress caused by defective SiSTL1 protein.

Finally, the ability of the S-phase checkpoint to prevent late replication-origin firing is mediated by ATR–CHK1-dependent phosphorylation of replication origin proteins (Giannattasio and Branzei, 2017). This regulation, however, is at the protein level. In this study, we have provided new perspectives on how defective SiSTL1 protein impedes DNA replication at the transcriptional level.

## Supplementary data

Supplementary data are available at *JXB* online.

Fig. S1. Density curves of BSCs/MCs containing zero to six chloroplasts in Yugu1 and *sistl1*.

Fig. S2. Photographs showing four striped T_0_ lines, striped T_1_ lines of *OsC1-4* and *OsC2-12*, and T_2_ lines of *OsC1-8*.

Fig. S3. Sequencing analysis of *OsC1-8*, *OsC1-4*, *OsC2-12*, and their descendants.

Fig. S4. Agarose gel electrophoresis for the PCR products amplified with *Cas9* gene-specific primers from the T_1_ and T_2_ descendants of *OsC1-4*, *OsC2-12*, and *OsC1-8*.

Fig. S5. GO term enrichment analysis for the 1082 cell cycle-regulated genes described by Menges *et al.* (2003) in Arabidopsis and 214 *Setaria* homologs differentially expressed between Yugu1 and *sistl1.*

Fig. S6. Verifying the relative expression level of *SiSTL1*, *SiSTL1-2*, *SiSTL1-3*, *E2F1*, and *E2F2* along leaf developmental gradients in Yugu1 and *sistl1* fourth leaves with semi-quantitative RT-PCR.

Fig. S7. Relative expression levels of *OsRNRL1*, *OsE2F1*, *OsCDK2*, *OsCycA1:1*, and *OsCycB1:1* in T_1_ descendants of *OsC1-4* and *OsC2-12*, and T_2_ descendants of *OsC1-8*.

Table S1. Locus and primer sequences of InDel and CAPS markers.

Table S2. Primers used for vectors construction.

Table S3. Primers used for and transgenic verification.

Table S4. Primers used for qRT-PCR.

Table S5. Verification of RNA-seq result by qRT-PCR.

Table S6. SNPs identified within the candidate interval (91 kb).

Table S7. Differentially expressed genes involved in DNA replication in *sistl1* and Yugu1.

Table S8. 486 cell cycle regulated genes expressed in *sistl1* and Yugu1.

Table S9. Genes differentially expressed in *sistl1* and Yugu1.

Supplementary Figures S1-S7

Supplementary Figure S8

Supplementary Tables S1-S3 and S6

Supplementary Table S4

Supplementary Table S5

Supplementary Table S7

Supplementary Table S8

Supplementary Table S9

## Data deposition

Data collected from the germination trials, flow cytometic analysis and expression changes on *sistl1* and Yugu1 are available at Dryad Digital Repository (https://doi.org/10.5061/dryad.t6v7t8s; Tang *et al*. 2018)
