# Supplementary material for: SiSTL1, encoding a large subunit of ribonucleotide reductase, is crucial for plant growth, chloroplast biogenesis, and cell cycle progression in Setaria italica
Source: J Exp Bot. 2018 Dec 7;70(4):1167–82. doi: 10.1093/jxb/ery429 (PMC6382339; doi:10.1093/jxb/ery429)
Supplement: Supplementary Figures S1-S7 [file ery429_suppl_supplementary_figures_1-7.pptx]

## Slide 1
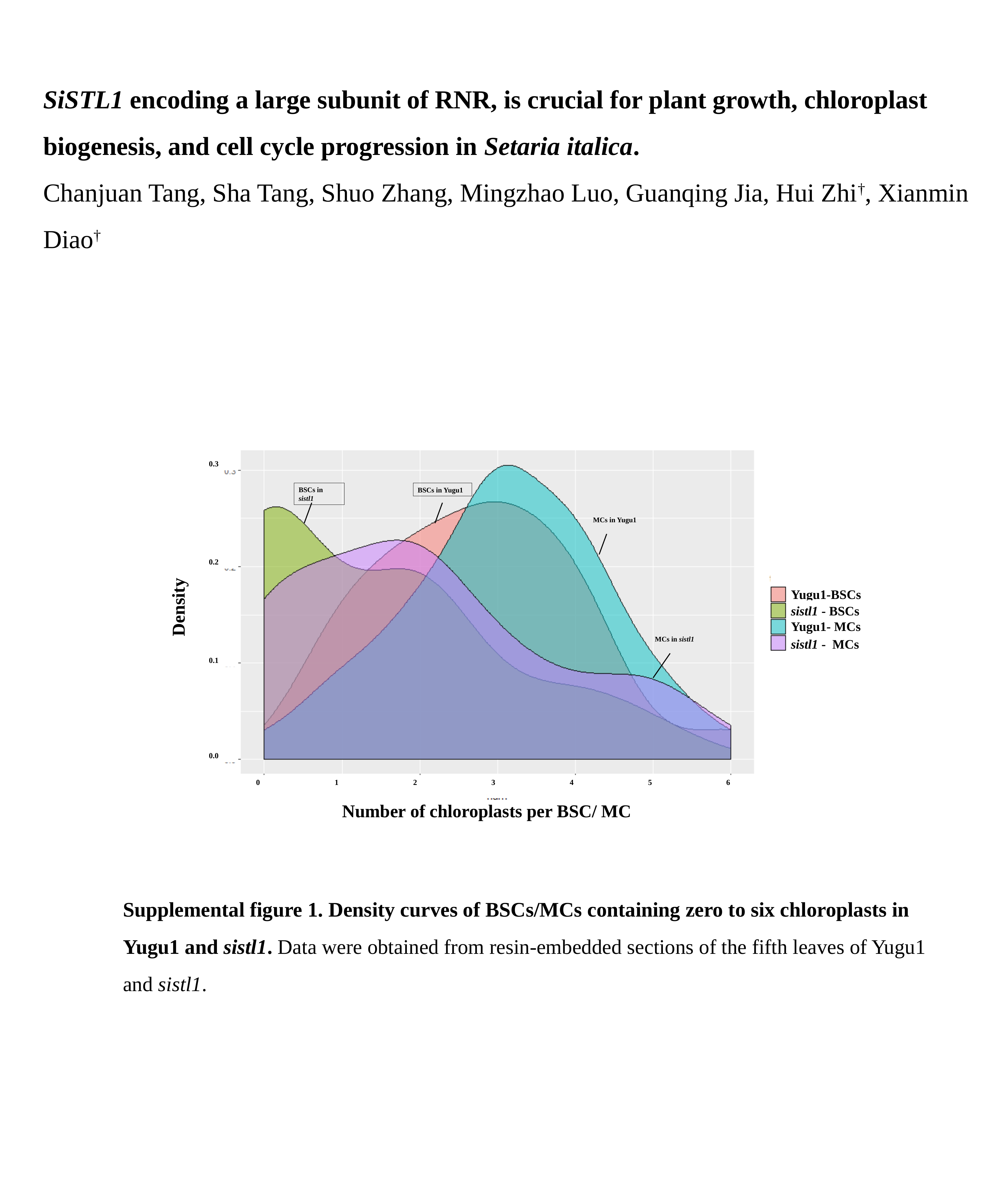

SiSTL1 encoding a large subunit of RNR, is crucial for plant growth, chloroplast biogenesis, and cell cycle progression in Setaria italica.
Chanjuan Tang, Sha Tang, Shuo Zhang, Mingzhao Luo, Guanqing Jia, Hui Zhi†, Xianmin Diao†
0.3
BSCs in sistl1
BSCs in Yugu1
MCs in Yugu1
0.2
Yugu1-BSCs
Density
sistl1 - BSCs
Yugu1- MCs
MCs in sistl1
sistl1 - MCs
0.1
0.0
5
6
0
1
2
3
4
Number of chloroplasts per BSC/ MC
Supplemental figure 1. Density curves of BSCs/MCs containing zero to six chloroplasts in Yugu1 and sistl1. Data were obtained from resin-embedded sections of the fifth leaves of Yugu1 and sistl1.

## Slide 2
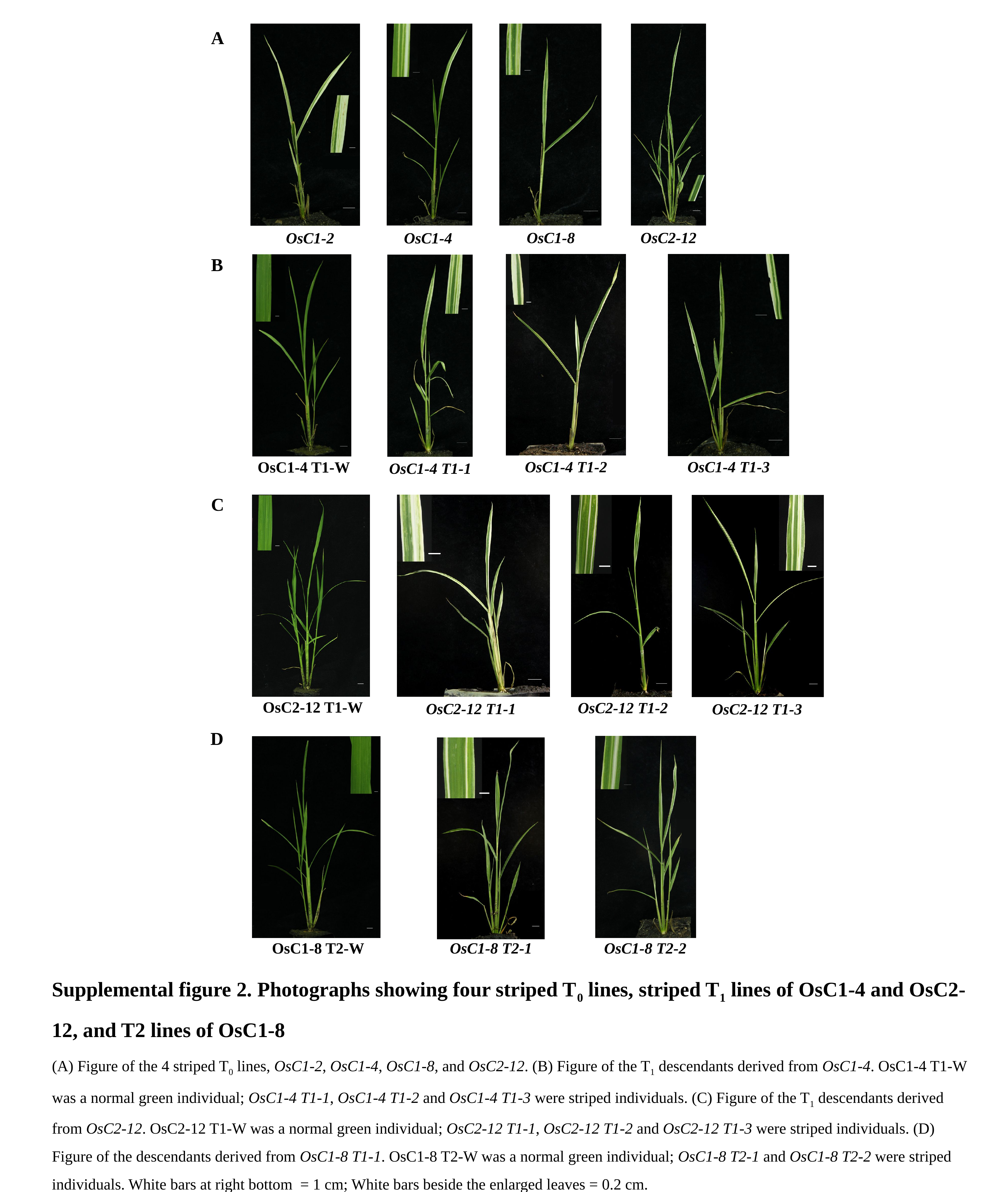

A
B
OsC1-4 T1-3
OsC1-4 T1-2
OsC1-4 T1-W
OsC1-4 T1-1
C
OsC2-12 T1-W
OsC2-12 T1-2
OsC2-12 T1-1
OsC2-12 T1-3
OsC1-8 T2-2
OsC1-8 T2-W
OsC1-8 T2-1
OsC2-12
OsC1-8
OsC1-4
OsC1-2
D
Supplemental figure 2. Photographs showing four striped T0 lines, striped T1 lines of OsC1-4 and OsC2-12, and T2 lines of OsC1-8
(A) Figure of the 4 striped T0 lines, OsC1-2, OsC1-4, OsC1-8, and OsC2-12. (B) Figure of the T1 descendants derived from OsC1-4. OsC1-4 T1-W was a normal green individual; OsC1-4 T1-1, OsC1-4 T1-2 and OsC1-4 T1-3 were striped individuals. (C) Figure of the T1 descendants derived from OsC2-12. OsC2-12 T1-W was a normal green individual; OsC2-12 T1-1, OsC2-12 T1-2 and OsC2-12 T1-3 were striped individuals. (D) Figure of the descendants derived from OsC1-8 T1-1. OsC1-8 T2-W was a normal green individual; OsC1-8 T2-1 and OsC1-8 T2-2 were striped individuals. White bars at right bottom = 1 cm; White bars beside the enlarged leaves = 0.2 cm.

## Slide 3
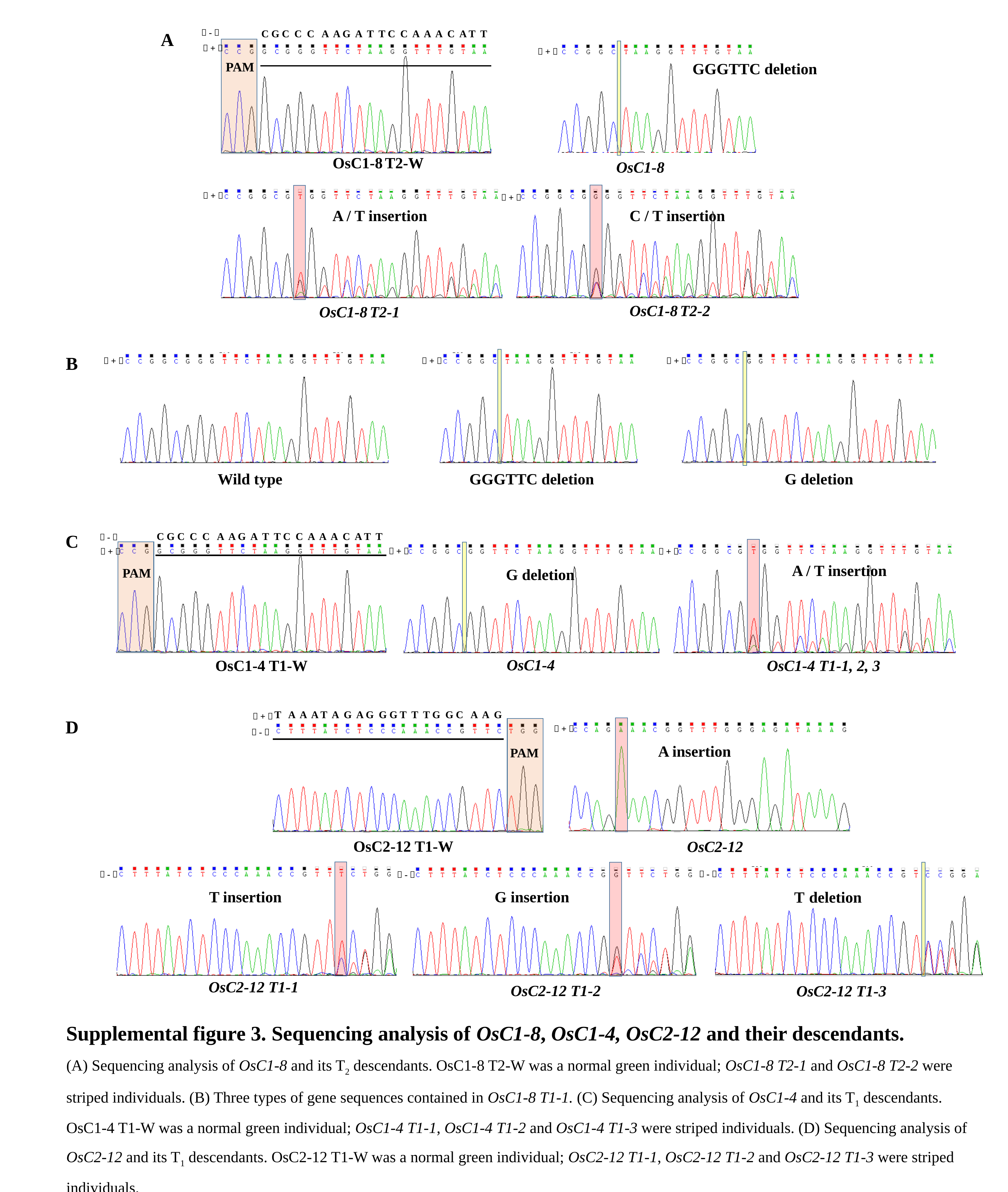

（-）
C G C C C A A G A T T C C A A A C A T T
（+）
PAM
A / T insertion
C / T insertion
GGGTTC deletion
OsC1-8
OsC1-8 T2-2
OsC1-8 T2-1
（+）
（+）
（+）
OsC1-8 T2-W
A
B
（+）
（+）
（+）
Wild type
G deletion
GGGTTC deletion
C
C G C C C A A G A T T C C A A A C A T T
（-）
（+）
A / T insertion
PAM
G deletion
OsC1-4
OsC1-4 T1-1, 2, 3
（+）
（+）
OsC1-4 T1-W
T A A A T A G A G G G T T T G G C A A G
（+）
PAM
T deletion
G insertion
（-）
（+）
A insertion
OsC2-12
T insertion
（-）
（-）
（-）
OsC2-12 T1-1
OsC2-12 T1-2
OsC2-12 T1-3
OsC2-12 T1-W
D
Supplemental figure 3. Sequencing analysis of OsC1-8, OsC1-4, OsC2-12 and their descendants.
(A) Sequencing analysis of OsC1-8 and its T2 descendants. OsC1-8 T2-W was a normal green individual; OsC1-8 T2-1 and OsC1-8 T2-2 were striped individuals. (B) Three types of gene sequences contained in OsC1-8 T1-1. (C) Sequencing analysis of OsC1-4 and its T1 descendants. OsC1-4 T1-W was a normal green individual; OsC1-4 T1-1, OsC1-4 T1-2 and OsC1-4 T1-3 were striped individuals. (D) Sequencing analysis of OsC2-12 and its T1 descendants. OsC2-12 T1-W was a normal green individual; OsC2-12 T1-1, OsC2-12 T1-2 and OsC2-12 T1-3 were striped individuals.

## Slide 4
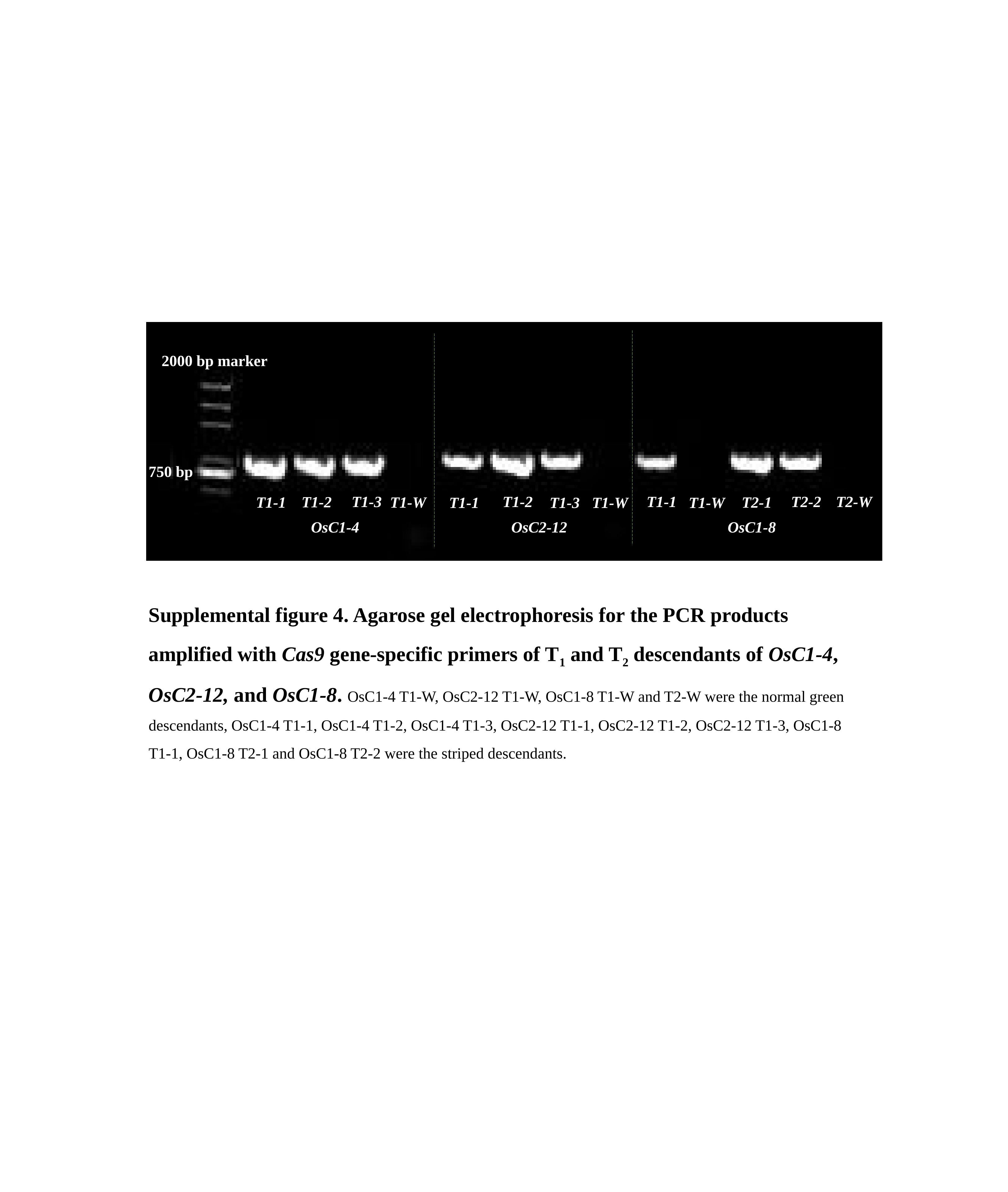

T2-2
T2-W
T1-2
T1-3
T1-2
T1-1
T1-W
T2-1
T1-1
T1-3
T1-W
OsC1-4
OsC2-12
OsC1-8
2000 bp marker
750 bp
T1-1
T1-W
Supplemental figure 4. Agarose gel electrophoresis for the PCR products amplified with Cas9 gene-specific primers of T1 and T2 descendants of OsC1-4, OsC2-12, and OsC1-8. OsC1-4 T1-W, OsC2-12 T1-W, OsC1-8 T1-W and T2-W were the normal green descendants, OsC1-4 T1-1, OsC1-4 T1-2, OsC1-4 T1-3, OsC2-12 T1-1, OsC2-12 T1-2, OsC2-12 T1-3, OsC1-8 T1-1, OsC1-8 T2-1 and OsC1-8 T2-2 were the striped descendants.

## Slide 5
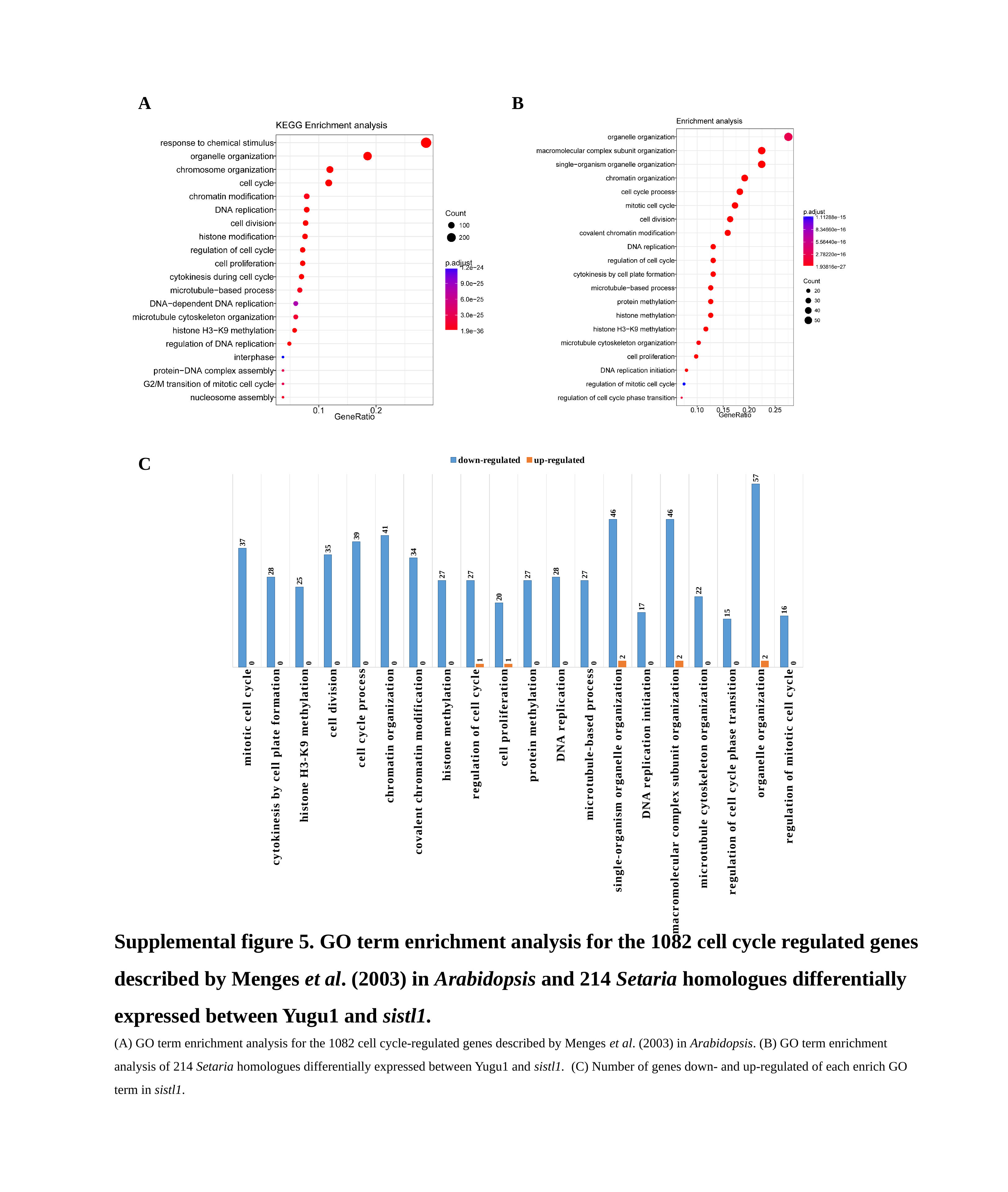

A
B
C
### Chart
| Category | down-regulated | up-regulated |
|---|---|---|
| mitotic cell cycle | 37.0 | 0.0 |
| cytokinesis by cell plate formation | 28.0 | 0.0 |
| histone H3-K9 methylation | 25.0 | 0.0 |
| cell division | 35.0 | 0.0 |
| cell cycle process | 39.0 | 0.0 |
| chromatin organization | 41.0 | 0.0 |
| covalent chromatin modification | 34.0 | 0.0 |
| histone methylation | 27.0 | 0.0 |
| regulation of cell cycle | 27.0 | 1.0 |
| cell proliferation | 20.0 | 1.0 |
| protein methylation | 27.0 | 0.0 |
| DNA replication | 28.0 | 0.0 |
| microtubule-based process | 27.0 | 0.0 |
| single-organism organelle organization | 46.0 | 2.0 |
| DNA replication initiation | 17.0 | 0.0 |
| macromolecular complex subunit organization | 46.0 | 2.0 |
| microtubule cytoskeleton organization | 22.0 | 0.0 |
| regulation of cell cycle phase transition | 15.0 | 0.0 |
| organelle organization | 57.0 | 2.0 |
| regulation of mitotic cell cycle | 16.0 | 0.0 |
Supplemental figure 5. GO term enrichment analysis for the 1082 cell cycle regulated genes described by Menges et al. (2003) in Arabidopsis and 214 Setaria homologues differentially expressed between Yugu1 and sistl1.
(A) GO term enrichment analysis for the 1082 cell cycle-regulated genes described by Menges et al. (2003) in Arabidopsis. (B) GO term enrichment analysis of 214 Setaria homologues differentially expressed between Yugu1 and sistl1. (C) Number of genes down- and up-regulated of each enrich GO term in sistl1.

## Slide 6
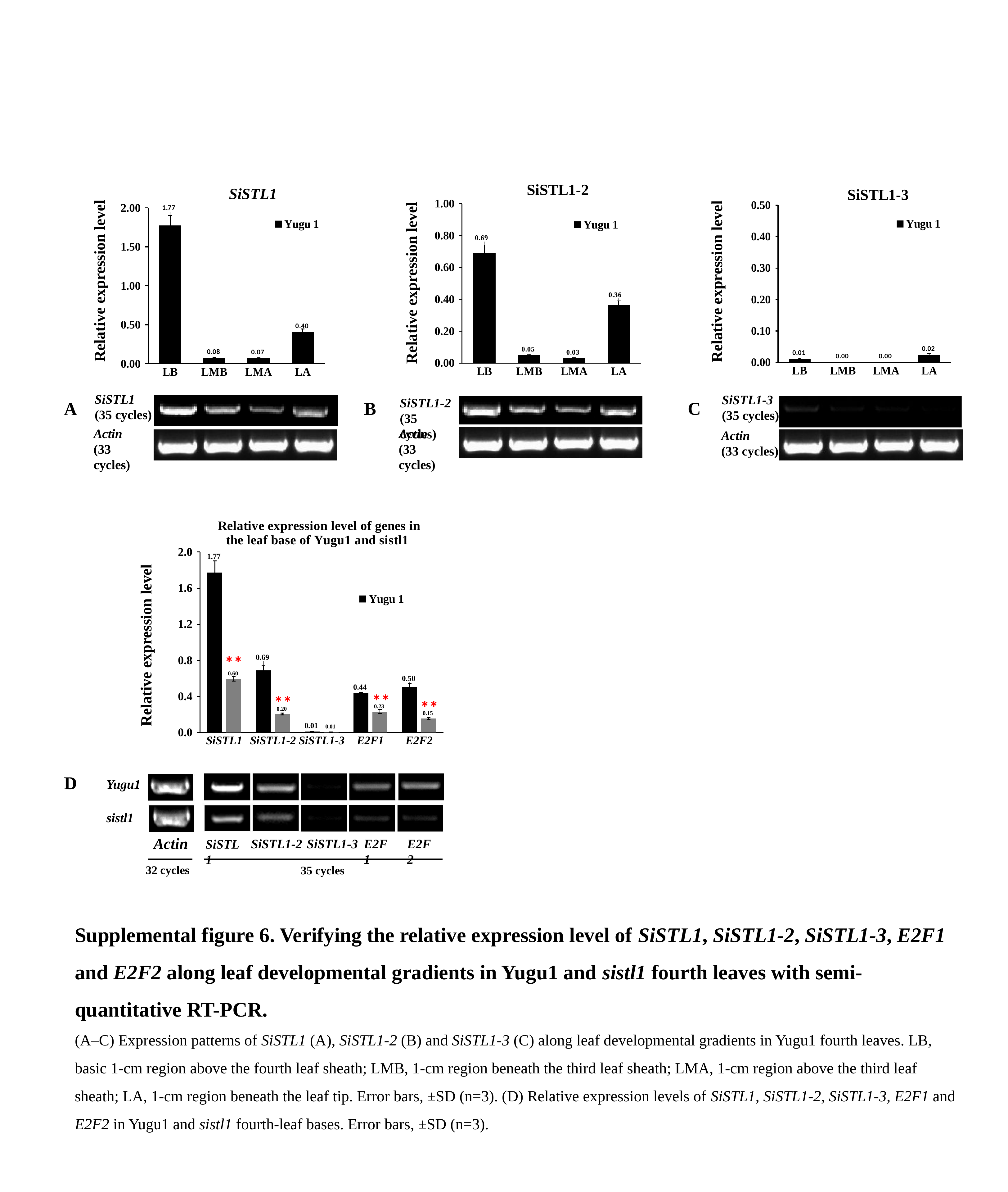

### Chart: SiSTL1-3
| Category | Yugu 1 |
|---|---|
| LB | 0.011451655613094013 |
| LMB | 0.0008709233259263523 |
| LMA | 0.000851031409670844 |
| LA | 0.024257365215576317 |
Relative expression level
SiSTL1-3
(35 cycles)
Actin
(33 cycles)
C
### Chart: SiSTL1-2
| Category | Yugu 1 |
|---|---|
| LB | 0.6895848575270108 |
| LMB | 0.05113896125074018 |
| LMA | 0.030676974179667447 |
| LA | 0.3643676209292329 |Relative expression level
SiSTL1-2
(35 cycles)
Actin
(33 cycles)
B
### Chart: SiSTL1
| Category | Yugu 1 |
|---|---|
| LB | 1.7733778978439165 |
| LMB | 0.07665019823918573 |
| LMA | 0.0719597175124988 |
| LA | 0.40493623942328816 |Relative expression level
SiSTL1
(35 cycles)
Actin
(33 cycles)
A
### Chart: Relative expression level of genes in the leaf base of Yugu1 and sistl1
| Category | Yugu 1 | sistl1 |
|---|---|---|
| SiSTL1 | 1.7733778978439165 | 0.5962819896073538 |
| SiSTL1-2 | 0.6895848575270108 | 0.2046882226384448 |
| SiSTL1-3 | 0.011451655613094013 | 0.006497798255286173 |
| E2F1 | 0.43737217110011617 | 0.23167913079373773 |
| E2F2 | 0.5028053346864687 | 0.15437959506409824 |Relative expression level
Yugu1
sistl1
SiSTL1-2
SiSTL1-3
Actin
E2F2
E2F1
SiSTL1
D
**
**
**
**
32 cycles
35 cycles
Supplemental figure 6. Verifying the relative expression level of SiSTL1, SiSTL1-2, SiSTL1-3, E2F1 and E2F2 along leaf developmental gradients in Yugu1 and sistl1 fourth leaves with semi-quantitative RT-PCR.
(A–C) Expression patterns of SiSTL1 (A), SiSTL1-2 (B) and SiSTL1-3 (C) along leaf developmental gradients in Yugu1 fourth leaves. LB, basic 1-cm region above the fourth leaf sheath; LMB, 1-cm region beneath the third leaf sheath; LMA, 1-cm region above the third leaf sheath; LA, 1-cm region beneath the leaf tip. Error bars, ±SD (n=3). (D) Relative expression levels of SiSTL1, SiSTL1-2, SiSTL1-3, E2F1 and E2F2 in Yugu1 and sistl1 fourth-leaf bases. Error bars, ±SD (n=3).

## Slide 7
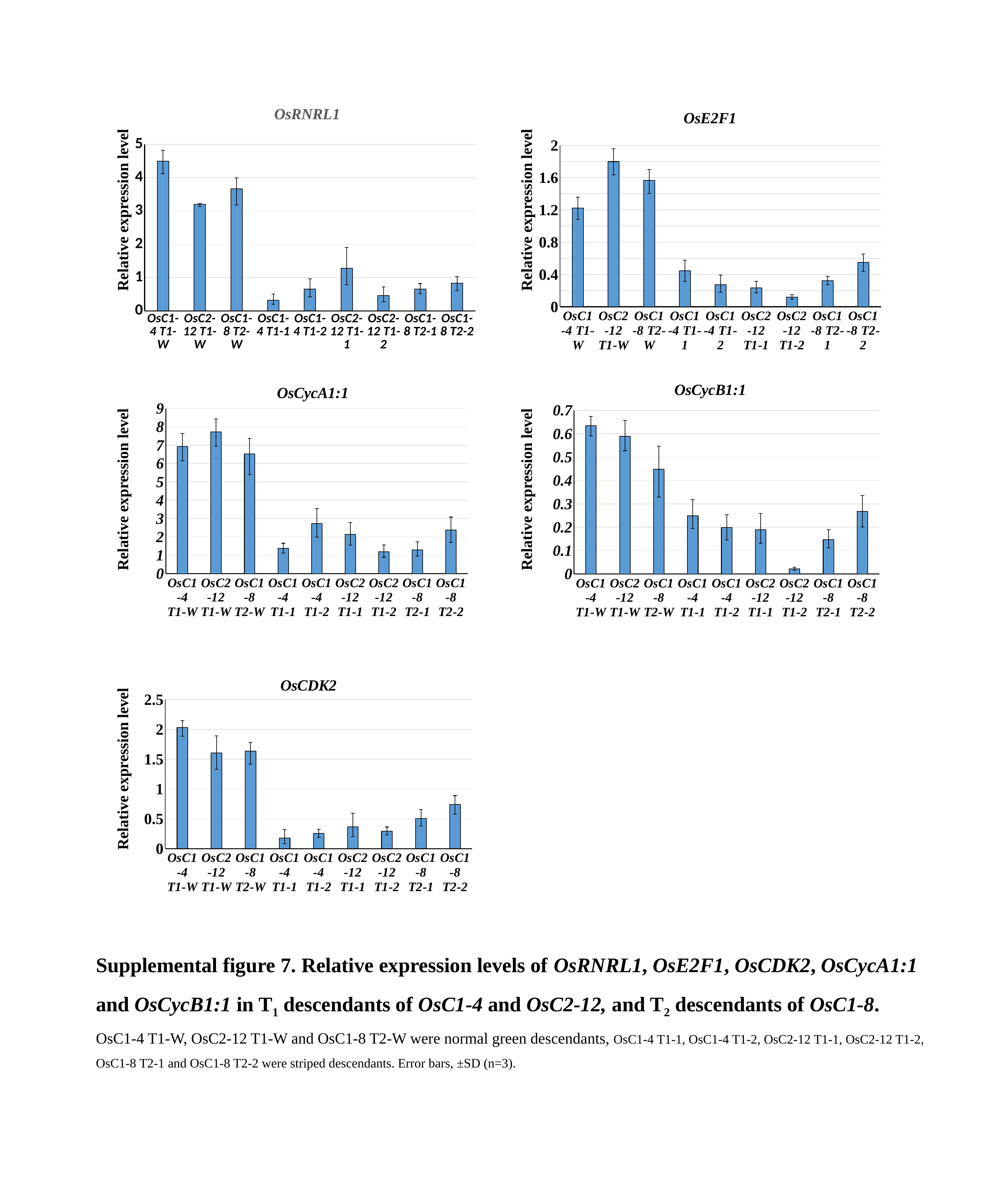

### Chart: OsRNRL1
| Category | OsRNRL1 |
|---|---|
| OsC1-4 T1-W | 4.491521892975082 |
| OsC2-12 T1-W | 3.196196350222486 |
| OsC1-8 T2-W | 3.668690394591081 |
| OsC1-4 T1-1 | 0.32224348440767925 |
| OsC1-4 T1-2 | 0.6505095261624321 |
| OsC2-12 T1-1 | 1.2781610773437933 |
| OsC2-12 T1-2 | 0.4555384432888519 |
| OsC1-8 T2-1 | 0.6502128805992359 |
| OsC1-8 T2-2 | 0.8267487334476017 |
### Chart: OsE2F1
| Category | OsE2F1 |
|---|---|
| OsC1-4 T1-W | 1.2242889550292 |
| OsC2-12 T1-W | 1.7980313966671753 |
| OsC1-8 T2-W | 1.5639929612413417 |
| OsC1-4 T1-1 | 0.44759738944208416 |
| OsC1-4 T1-2 | 0.2725565514404011 |
| OsC2-12 T1-1 | 0.23435183507408466 |
| OsC2-12 T1-2 | 0.11875220026804902 |
| OsC1-8 T2-1 | 0.3224553011553984 |
| OsC1-8 T2-2 | 0.5503035371764864 |Relative expression level
Relative expression level
### Chart: OsCycB1:1
| Category | OsCycB1:1 |
|---|---|
| OsC1-4 T1-W | 0.634191392699493 |
| OsC2-12 T1-W | 0.5892708083942483 |
| OsC1-8 T2-W | 0.44921318535592986 |
| OsC1-4 T1-1 | 0.24919093417132987 |
| OsC1-4 T1-2 | 0.19835449878095532 |
| OsC2-12 T1-1 | 0.1891558456608057 |
| OsC2-12 T1-2 | 0.021572193192733655 |
| OsC1-8 T2-1 | 0.14640386601324884 |
| OsC1-8 T2-2 | 0.2683814361133222 |
### Chart: OsCycA1:1
| Category | OsCycA1:1 |
|---|---|
| OsC1-4 T1-W | 6.934449918927328 |
| OsC2-12 T1-W | 7.726197054602774 |
| OsC1-8 T2-W | 6.525327335748443 |
| OsC1-4 T1-1 | 1.377022136304884 |
| OsC1-4 T1-2 | 2.7382925052542597 |
| OsC2-12 T1-1 | 2.1416496947173687 |
| OsC2-12 T1-2 | 1.185110560450444 |
| OsC1-8 T2-1 | 1.2923726780095361 |
| OsC1-8 T2-2 | 2.382249830157799 |Relative expression level
Relative expression level
### Chart: OsCDK2
| Category | OsCDK2 |
|---|---|
| OsC1-4 T1-W | 2.0312399766225364 |
| OsC2-12 T1-W | 1.6035439478172024 |
| OsC1-8 T2-W | 1.6362383670633402 |
| OsC1-4 T1-1 | 0.18070751295721324 |
| OsC1-4 T1-2 | 0.2554703678283703 |
| OsC2-12 T1-1 | 0.366027304181397 |
| OsC2-12 T1-2 | 0.2926009941681625 |
| OsC1-8 T2-1 | 0.5083038828820791 |
| OsC1-8 T2-2 | 0.7403364268283213 |Relative expression level
Supplemental figure 7. Relative expression levels of OsRNRL1, OsE2F1, OsCDK2, OsCycA1:1 and OsCycB1:1 in T1 descendants of OsC1-4 and OsC2-12, and T2 descendants of OsC1-8.
OsC1-4 T1-W, OsC2-12 T1-W and OsC1-8 T2-W were normal green descendants, OsC1-4 T1-1, OsC1-4 T1-2, OsC2-12 T1-1, OsC2-12 T1-2, OsC1-8 T2-1 and OsC1-8 T2-2 were striped descendants. Error bars, ±SD (n=3).
