# Supplementary material for: SiSTL1, encoding a large subunit of ribonucleotide reductase, is crucial for plant growth, chloroplast biogenesis, and cell cycle progression in Setaria italica
Source: J Exp Bot. 2018 Dec 7;70(4):1167–82. doi: 10.1093/jxb/ery429 (PMC6382339; doi:10.1093/jxb/ery429)
Supplement: Supplementary Figure S8 [file ery429_suppl_supplementary_supplementary_figures_8.pdf]

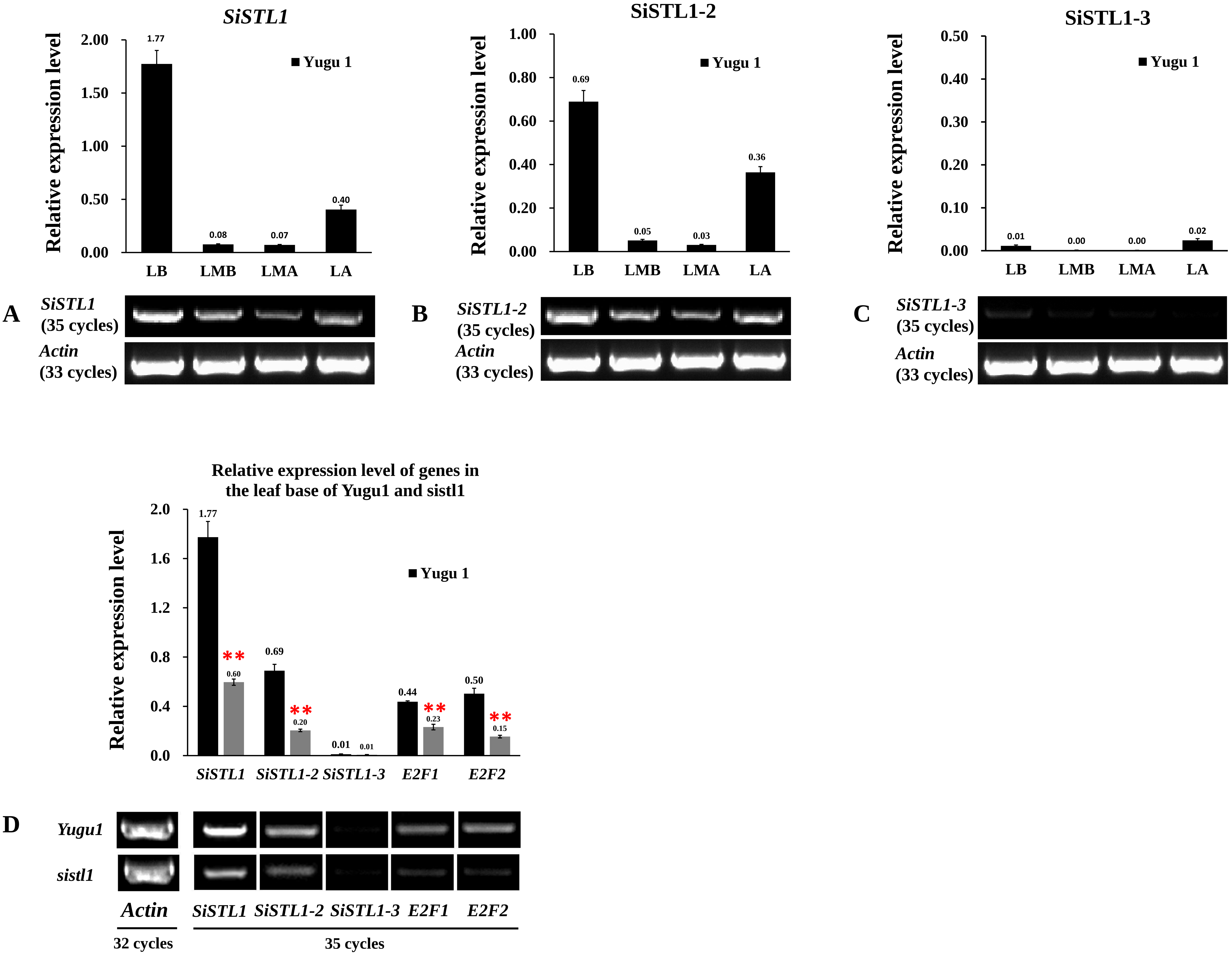

Supplemental figure 8. Verifying the relative expression level of *SiSTL1*, *SiSTL1-2*, *SiSTL1-3*, *E2F1* and *E2F2* along leaf developmental gradients in Yugu1 and *sistl1* fourth leaves with semi-quantitative RT-PCR.

(A–C) Expression patterns of *SiSTL1* (A), *SiSTL1-2* (B) and *SiSTL1-3* (C) along leaf developmental gradients in Yugu1 fourth leaves. LB, basic 1-cm region above the fourth leaf sheath; LMB, 1-cm region beneath the third leaf sheath; LMA, 1-cm region above the third leaf sheath; LA, 1-cm region beneath the leaf tip. Error bars, ±SD (n=3). (D) Relative expression levels of *SiSTL1*, *SiSTL1-2*, *SiSTL1-3*, *E2F1* and *E2F2* in Yugu1 and *sistl1* fourth-leaf bases. Error bars, ±SD (n=3).
