# Supplementary material for: SiSTL1, encoding a large subunit of ribonucleotide reductase, is crucial for plant growth, chloroplast biogenesis, and cell cycle progression in Setaria italica
Source: J Exp Bot. 2018 Dec 7;70(4):1167–82. doi: 10.1093/jxb/ery429 (PMC6382339; doi:10.1093/jxb/ery429)
Supplement: Supplementary Tables S1-S3 and S6 [file ery429_suppl_supplementary_tables_s1-s3_s6.docx]

**Supplementary table 1. Locus and primer sequences of InDel and CAPS markers.**

| **Primer name** | **Location** | **Forward primer sequence** | **Reverse primer sequence** |
| --- | --- | --- | --- |
| InDel 14 | 4232606 | TACTCATTGCATCCCCTTCAGCAGC | CACTGGATAACGCATGGACTGACTA |
| InDel 1420-2 | 4335413 | GGAGAACTTTTAGCTTCAGTAG | CCTGTGATTATGGTTGGTC |
| InDel 004-3 | 4479785 | TGACCTAAGTGATACAGCGAAAC | CACCAAACAATTAACCAAATGAA |
| InDel 1422-2 | 5381211 | ATAGTAATTGGCCCATCAC | AGGGAGGAGAAGGTCATAC |
| CAPS-8 | 4339573 | CCTGCAACCCGCCATAC | CGTCTCAAATTCGCAACCA |
| CAPS-7 | 4430449 | GGAGGTCTCATCTAGATCCG | CATCCTAGTTCCGGTACTCT |

**Supplementary table 2. Primers used for vectors construction**

| **Primer name** | **primer sequence** |
| --- | --- |
| gROsT1 | TTACAAACCTTAGAACCCGCGTTTTAGAGCTAGAAAT |
| gROsT2 | CTTTATCTCCCAAACCGTTCGTTTTAGAGCTAGAAAT |
| U6aOsT1 | GCGGGTTCTAAGGTTTGTAACGGCAGCCAAGCCAGCA |
| U6aOsT2 | GAACGGTTTGGGAGATAAAGCGGCAGCCAAGCCAGCA |
| U-F | CTCCGTTTTACCTGTGGAATCG |
| gR-R | CGGAGGAAAATTCCATCCAC |
| U-GAL | ACCGGTAAGGCGCGCCGTAGTGCTCGACTAGTATGGAATCGGCAGCAAAGG |
| Pgs-GAR | TAGCTCGAGAGGCGCGCCAATGATACCGACGCGTATCCATCCACTCCAAGCTCTTG |
| hGFP F | TATCTCTAGAGGATCCCTTCCGACCTCCCTTCC |
| hGFP R | TGCTCACCATGGATCCGCTTCCGCACGCCAGGCAT |
| Yeast-AD-SiSTL1 F | CATCGATACGGGATCCATATGTACGTGGTGAAGCGTGAC |
| Yeast-AD- SiSTL 1 R | CGAGCTCGATGGATCCCCTACATCACAGAC TTCGTTC |
| Yeast-BD-RNRS F | GAATTCCCGGGGATCCGTATGCCTGCCGCGCCGACG |
| Yeast-BD-RNRS R | GCAGGTCGA CGGATCCGAACA AGCAGGGTGTATTGAG |

**Supplementary table 3. Primers used for and transgenic verification.**

| **Primer name** | **primer sequence** |
| --- | --- |
| crispr V3 F | ATCAGGGATAAGCAGAGC |
| crispr V3 R | GAGCGTGGTGGTAGTTGT |
| OsCas9-C F | GGGCATTCTCCAACCTGA |
| OsCas9-C R | CATGGCATGACAAATACAGA |

**Supplemental table 6. SNPs identified within the candidate interval (91-kb).**

| **POS** | **REF** | **ALT** | **DP4** | **INFO** |
| --- | --- | --- | --- | --- |
| Chr4: 4347392 | G | A | DP4=0,0,20,24 | *Seita.4G058800*, exon, non-synonymous mutation, "Gly" to "Glu" |
| Chr4: 4412036 | G | A | DP4=0,0,18,14 | Intergenic |
| Chr4: 4429538 | A | G | DP4=0,0,5,23 | Background SNP that also was found in other *Setaria* mutants, “*SiDWARF3*” and “*Loose Panicle1* ” |
